# Spindle-F Is the Central Mediator of Ik2 Kinase-Dependent Dendrite Pruning in *Drosophila* Sensory Neurons

**DOI:** 10.1371/journal.pgen.1005642

**Published:** 2015-11-05

**Authors:** Tzu Lin, Po-Yuan Pan, Yu-Ting Lai, Kai-Wen Chiang, Hsin-Lun Hsieh, Yi-Ping Wu, Jian-Ming Ke, Myong-Chol Lee, Shih-Sian Liao, Hsueh-Tzu Shih, Chiou-Yang Tang, Shi-Bing Yang, Hsu-Chen Cheng, June-Tai Wu, Yuh-Nung Jan, Hsiu-Hsiang Lee

**Affiliations:** 1 Institute of Molecular Medicine, College of Medicine, National Taiwan University, Taipei, Taiwan; 2 Institute of Molecular Biology, Academia Sinica, Taipei, Taiwan; 3 Howard Hughes Medical Institute, Department of Physiology, Biochemistry and Biophysics, University of California, San Francisco, San Francisco, California, United States of America; 4 Department of Life Sciences, National Chung Hsing University, Taichung, Taiwan; Weizmann Institute, ISRAEL

## Abstract

During development, certain *Drosophila* sensory neurons undergo dendrite pruning that selectively eliminates their dendrites but leaves the axons intact. How these neurons regulate pruning activity in the dendrites remains unknown. Here, we identify a coiled-coil protein Spindle-F (Spn-F) that is required for dendrite pruning in *Drosophila* sensory neurons. Spn-F acts downstream of IKK-related kinase Ik2 in the same pathway for dendrite pruning. Spn-F exhibits a punctate pattern in larval neurons, whereas these Spn-F puncta become redistributed in pupal neurons, a step that is essential for dendrite pruning. The redistribution of Spn-F from puncta in pupal neurons requires the phosphorylation of Spn-F by Ik2 kinase to decrease Spn-F self-association, and depends on the function of microtubule motor dynein complex. Spn-F is a key component to link Ik2 kinase to dynein motor complex, and the formation of Ik2/Spn-F/dynein complex is critical for Spn-F redistribution and for dendrite pruning. Our findings reveal a novel regulatory mechanism for dendrite pruning achieved by temporal activation of Ik2 kinase and dynein-mediated redistribution of Ik2/Spn-F complex in neurons.

## Author Summary

In *Drosophila*, the nervous systems undergo extensive neuronal remodeling during metamorphosis, as many larval neurons die and adult neurons are generated while some larval neurons survive and prune their branches. Pruning that removes specific parts of neuronal branches without causing cell death is a self-destruct process, thus requiring precise regulation to prevent undesired damage to the nervous systems. Certain *Drosophila* sensory neurons that undergo dendrite pruning, specifically eliminating the dendrites but leaving the axons intact, provide us an opportunity to study the mechanism of how pruning activity is regulated in the dendrites. We reasoned that the distinctive microtubule polarity in dendrites and axons might be involved and factors are required to regulate the pruning activity in the dendrites through their interaction with microtubules. Here, we identified Spindle-F that mediates Ik2-dependent pruning activity in the dendrites by linking Ik2 to the microtubule motor dynein complex. We showed that elevation of Ik2 activity during dendrite pruning promotes Ik2/Spindle-F/dynein complex moving along the microtubules. We also showed that the formation and redistribution of Ik2/Spindle-F/dynein complex are essential for dendrite pruning. Our study reveals a connection between the polarized microtubules of dendrites and the pruning activity through Spindle-F for dendrite pruning.

## Introduction

The precise assembly of neural circuits is crucial for the nervous system to function properly. The developing nervous systems often start with a primitive prototype, characterized by exuberant branches and excessive connections. Thus, further remodeling is required to refine the developing nervous systems to maturity. Neuronal pruning, one such remodeling mechanism, is a highly regulated self-destruct process that eliminates excessive neuronal branches in the absence of cell death. Pruning is widely observed in the nervous systems of both vertebrates and invertebrates [1,2], that not only ensures precise wiring during development, but also allows for adjustment of neuronal connections in response to injury and disease. Various studies have shown that defects in developmental pruning affect the function of the nervous systems in *C*. *elegans* [3] and *Drosophila* [4]. Moreover, a progressive loss of neurites far ahead of cell death is commonly observed in many neurodegenerative disorders [5,6]. Thus, any dysregulation of pruning activity even at the level of individual neurons would bring catastrophic consequences to the nervous systems. Although the primary triggers for developmental pruning and pruning that ensues upon neuronal injury and disease are diverse, the downstream machinery that eliminates neuronal processes shared some common features. For example, microtubule disruption is the earliest cellular event observed in all types of pruning [2,7,8], and the ubiquitin-proteasome system is required in all circumstances [7–10].

During *Drosophila* metamorphosis, substantial neuronal remodeling takes place in both the central and peripheral nervous systems [11–14]. Most of the larval peripheral neurons die during metamorphosis, whereas few, including some class IV dendritic arborization (C4da) neurons, survive and undergo large-scale dendrite pruning [13,14]. Dendrite pruning of the dorsal C4da neuron ddaC starts with severing of the proximal dendrites at 4–6 h APF (after puparium formation) [15]. Subsequently these disconnected dendrites become fragmented and eventually eliminated by the surrounding epidermal cells [16] by 16–18 h APF. In contrast to the central brain mushroom body (MB) γ neurons where both larval dendrites and axons are pruned during development, the peripheral C4da neurons specifically prune their dendrites keeping the axons intact [14]. The molecular basis for how the pruning activity is confined to the dendrites of C4da neurons remains unknown. We reasoned that molecular differences between dendrites and axons should be considered for such differential pruning activity in C4da neurons. It is known that microtubule polarity is different in the dendrites and axons of neurons [17], including in the *Drosophila* sensory neurons [18]. For example, C4da neurons have polarized microtubules in their proximal dendrites predominantly with microtubule minus end pointing away from the cell body, but have an opposite polarity in their axons [18,19]. This difference in microtubule polarity is essential for maintaining the proper function and compartmental identities of dendrites and axons, and might be an important determinant for spatially restricting pruning activity in the dendritic compartments of C4da neurons. Based on this assumption, some molecules are required to connect the pruning activity with the distinctive microtubule polarity of the dendrites in C4da neurons during dendrite pruning.

Previous studies have shown that dendrite pruning in C4da neurons is initiated by the steroid hormone ecdysone and its heterodimeric receptors, ecdysone receptor B1 (EcR-B1) and Ultraspiricle (Usp) [13,14]. Through transcriptional regulation of *sox14*, ecdysone signaling activates the Sox14 target gene *mical*, which encodes a cytoskeletal regulator, to regulate dendrite pruning [20]. A few other molecules mediating specific cellular activities have been shown to participate in dendrite pruning of C4da neurons, such as the ubiquitin-proteasome system [14], caspases [21,22], matrix metalloproteases [14], microtubule severing proteins [15] and mediators of dendritic calcium transients [23]. Our previous studies identified Ik2 kinase, a homologue of vertebrate IKK- ε ιν *Drosophila*, that plays an essential role in dendrite pruning of pupal neurons, and further demonstrated that Ik2 is sufficient to induce precocious dendrite severing in larval neurons [15]. To our knowledge, Ik2 is the only known molecule sufficient to induce premature dendrite severing in larvae, reflecting a central role of Ik2 kinase in dendrite pruning. Therefore, in this study we aimed to elucidate the mechanism by which Ik2 kinase signaling is transduced and regulated in *Drosophila* sensory neurons during dendrite pruning.

As Ik2 is essential for dendrite pruning, to elucidate the mechanism of Ik2 kinase signaling, we searched for candidate molecules that mediate Ik2 signals during dendrite pruning. Several lines of evidence suggested that Spn-F, a coil-coiled protein, is a good candidate. Firstly, *spn-F* mutant flies showed defects in developing oocytes and bristles [24], similar to the phenotypes observed in *ik2* mutants [25]. Secondly, Spn-F physically interacts with Ik2 [26]. It implied that *ik2* and *spn-F* may act in the same pathway during oogenesis and bristle morphogenesis, and raised the possibility that a similar pathway might also be involved in dendrite pruning of C4da neurons. Here, we demonstrate Spn-F playing a key role in linking Ik2 kinase to microtubule motor dynein complex for dendrite pruning. Spn-F acts downstream of Ik2 kinase in the same pathway for dendrite pruning. We show that Spn-F displays a punctate pattern in larval neurons and these Spn-F puncta become dispersed in pupal cells. The redistribution of Spn-F from puncta is essential for dendrite pruning, and depends on the activity of Ik2 kinase and the function of microtubule motor dynein complex. Our data also demonstrate that Spn-F not only links Ik2 to dynein motor complex, but also mediates the formation of Ik2/Spn-F/dynein complex, that is critical for Spn-F punctum disassembly and dendrite pruning.

## Results

### *spn-F* is required for dendrite pruning of *Drosophila* sensory neurons

**Fig 1:**
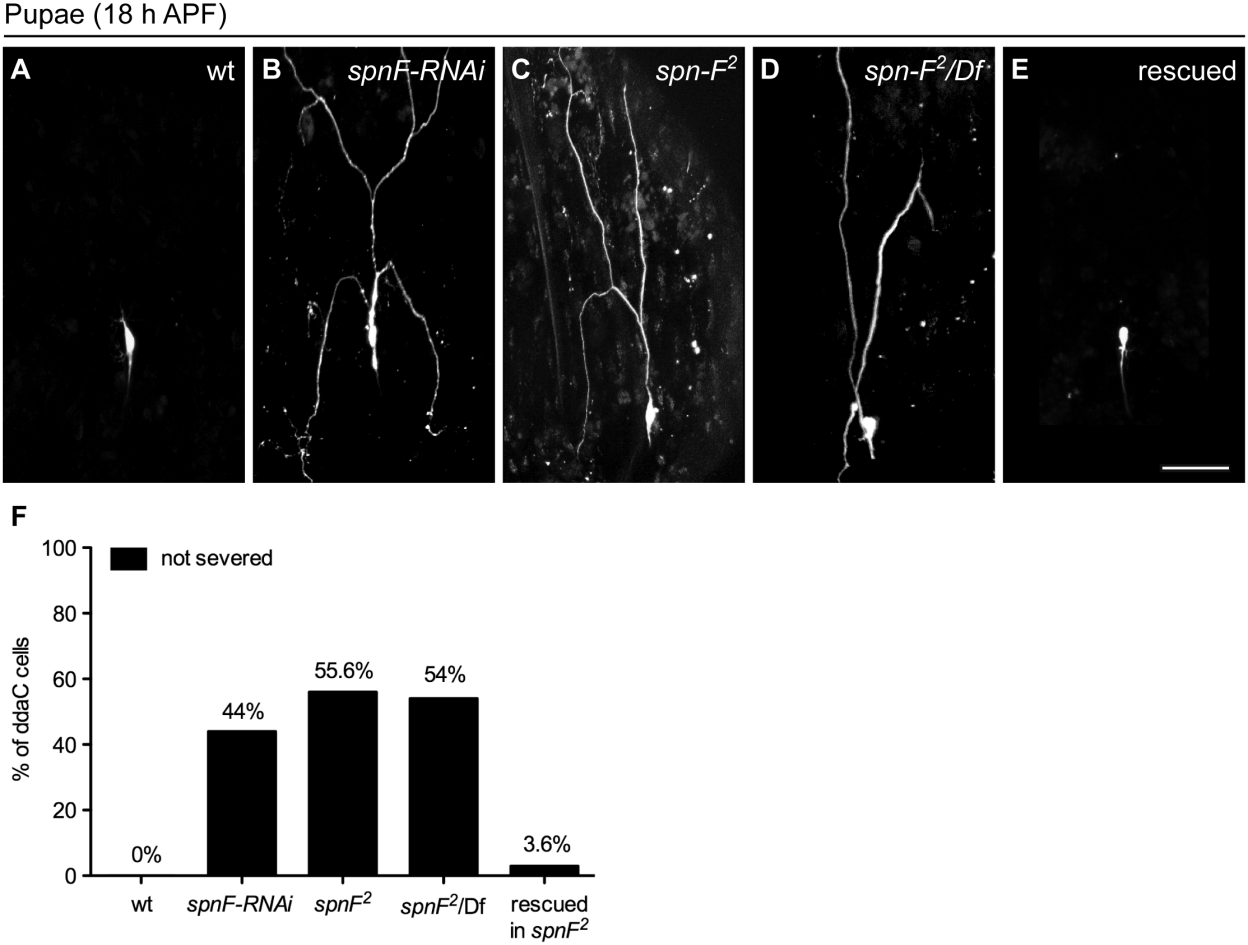
Spn-F is required for dendrite pruning. (A-E) The ddaC neurons are labeled with eGFP driven by *ppk* promoter (*ppk-eGFP*). At 18 h APF, the dendrites were severed and eliminated in wild-type ddaC neurons (A), but remained attached to the neurons of *spn-F-RNAi* mutants (B), of homozygous *spn-F* mutants (*spn-F* ^*2*^) (C), and of *spn-F*/*Df* mutants, which carry *spn-F* ^*2*^ on one chromosome and a deficiency *Df(3R)tll-e* (deletion includes the *spn-F* gene) on the other chromosome (D). (E) Dendrite pruning was observed normal in ddaC neurons of *spn-F* ^*2*^ mutants rescued with wild-type *spn-F-GFP* expression directed by *ppk-GAL4*. (F) Quantitative analysis of dendrite pruning phenotypes in ddaC cells at 18 h APF. The percentage of cells was determined by dividing the number of ddaC neurons with defective pruning by the total number of cells examined for each genotype; for wild type (wt), n = 50; for *spn-F-RNAi*, n = 100; for *spn-F* ^*2*^ mutants, n = 115; for *spn-F* ^*2*^ */Df*, n = 90; for *spn-F* ^*2*^ mutants rescued with wild-type *spn-F-GFP*, n = 88. Scale bar, 50 μm.

To examine the role of *spn-F* in dendrite pruning, we expressed *spn-F* double-strand RNAs (dsRNAs) under the control of class IV-specific *ppk-GAL4* [27] to reduce endogenous Spn-F expression. By 18 h APF wild-type neurons have pruned their dendrites (Fig 1A), however the primary dendrites remained connected to the cell body of C4da neurons with *spn-F* RNAi (RNA interference) (Fig 1B). We observed a similar phenotype in *spn-F* loss-of-function mutants (*spn-F* ^*2*^, [24]) (Fig 1C). Dendrite severing was likewise suppressed in neurons of *spn-F* ^*2*^ */Df* mutants (Fig 1D), which carry the *spn-F* ^*2*^ allele and a deficiency uncovering the entire *spn-F* gene locus. To confirm that these pruning defects were due to loss of *spn-F* from C4da neurons, we expressed the full-length *spn-F-GFP* directed by *ppk-GAL4* in *spn-F* ^*2*^ mutants and found that the impaired dendrite pruning was rescued (Fig 1E and 1F). Moreover, we also examined the dendritic morphology of larval C4da neurons of *spn-F* and *ik2* mutants, and found that dendrites develop normally in both mutant neurons (S1 Fig) [15], suggesting that the pruning defects observed in both mutants are not secondary to abnormal dendrite development. Taken together, these results indicated that *spn-F* is required for dendrite pruning in C4da neurons.

### *spn-F* acts downstream of *ik2* in dendrite pruning

**Fig 2:**
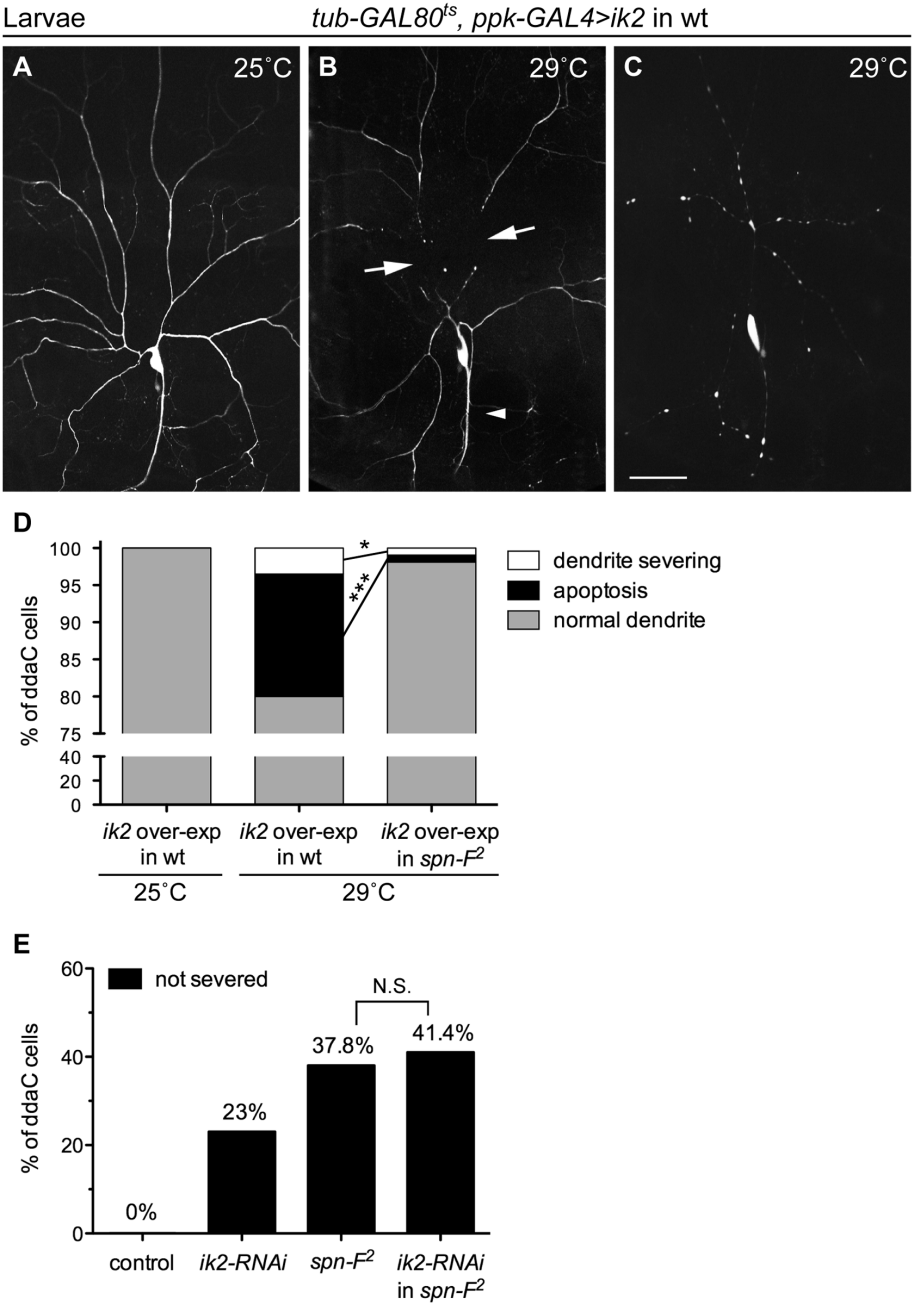
Spn-F acts downstream of Ik2 in dendrite pruning. The temporally increasing Ik2 activity in ddaC neurons of wild-type larvae (A-C) was controlled by the temperature-sensitive GAL80 (GAL80^ts^). Each animal contained the following transgenes: *tub-GAL80* ^*ts*^, *ppk-eGFP*, *ppk-GAL4*, and *UAS-ik2*. The dendrites of larval ddaC neurons displayed normal morphology at 25°C (A), but showed precocious dendrite-severing (arrows) at non-permissive temperature 29°C (B). Cell deaths were also observed in neurons at 29°C, as indicated by blebbing dendrites and axons (C). (D) Quantitative analysis of precocious dendrite-severing phenotypes and cell deaths observed in ddaC cells of wild-type and of *spn-F* ^*2*^ mutant larvae, which have *ik2* overexpression under the control of GAL80^ts^. In the wild-type larvae, the dendrites of all ddaC neurons showed normal morphology at 25°C (n = 100), but showed precocious dendrite-severing and apoptosis at 29°C (n = 200). Both precocious dendrite severing and apoptosis were suppressed in *spn-F* ^*2*^ mutant larvae at 29°C (n = 420). *, p = 0.0158; ***, p <0.0001. Statistical analysis was performed with Fisher’s exact test. (E) Quantitative analysis of dendrite pruning phenotypes in ddaC cells at 16 h APF. The percentage of cells was determined by dividing the number of ddaC neurons with defective pruning by the total number of cells examined for each genotype; for wild type (wt), n = 50; for *ik2-RNAi*, n = 100; for *spn-F* ^*2*^ mutants, n = 90; for *ik2-RNAi* in *spn-F* ^*2*^ mutants, n = 70. Statistical analysis was performed with Chi-square test. N.S., not significant. Scale bar, 50 μm.

Since *ik2* and *spn-F* mutant neurons displayed the same phenotype, we hypothesized that both genes function in the same pathway during dendrite pruning. To test this hypothesis, we performed genetic analyses between *ik2* and *spn-F*. It was known that *ik2* over-expression in larval C4da neurons causes cell death [15]. To avoid excessive apoptosis, we employed *ppk-GAL4* coupled with its temperature-sensitive inhibitor GAL80^ts^ to achieve spatial and temporal *ik2* expression in larval neurons by shifting temperature. Consistent with previous studies [15], no abnormality was detected in larval neurons under permissive temperature 25°C (Fig 2A). After shifting to non-permissive temperature 29°C, *ik2* overexpression not only triggered precocious dendrite severing (Fig 2B), but also caused apoptosis (Fig 2C) in wild-type larval neurons. Interestingly, we found that both precocious dendrite severing and apoptosis caused by *ik2* overexpression were significantly suppressed in neurons of *spn-F* mutants (Fig 2D), suggesting that *spn-F* functions downstream (or in parallel) of *ik2* in dendrite pruning. Additionally, we found no significant difference between the dendrite pruning defects observed in *spn-F* ^*2*^ mutants and that in *spn-F* ^*2*^ mutants with *ik2* RNAi (Fig 2E), indicating that both *ik2* and *spn-F* act in the same pathway of dendrite pruning. Together, our findings suggested that *spn-F* acts downstream of *ik2* in the same pathway during dendrite pruning.

### The distribution of Spn-F proteins depends on Ik2 kinase activity

**Fig 3:**
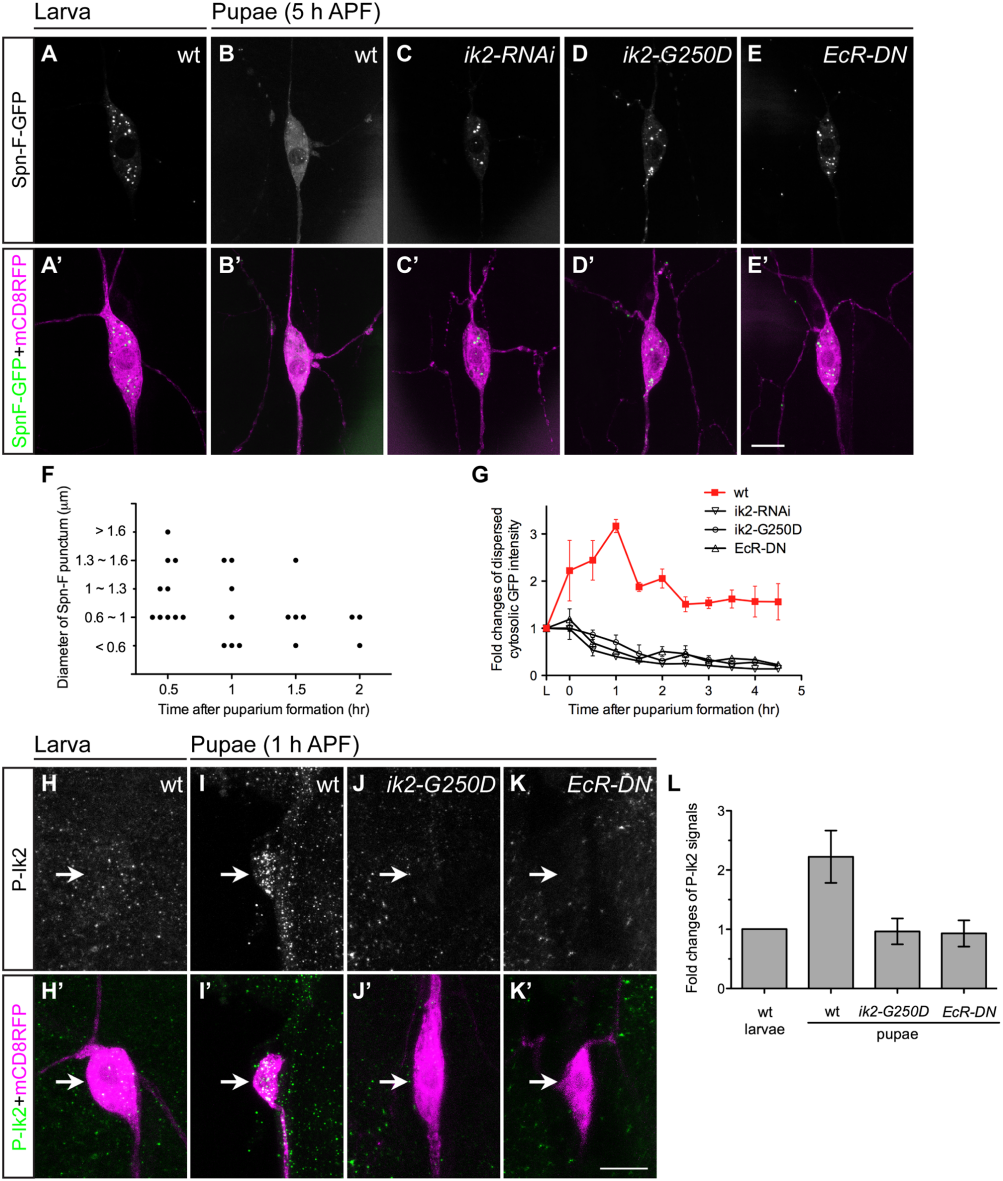
Ik2 kinase activity regulates the distribution of Spn-F in neurons. (A'-E', H'-K') The ddaC neurons were identified by *ppk-GAL4* and *UAS-mCD8RFP*. (A-E) The expression of *UAS-Spn-F-GFP* was controlled by *ppk-GAL4* in C4da neurons. The Spn-F-GFP showed punctate patterns in the cytosol of ddaC neurons of wild-type larvae (A). At 5 h APF, Spn-F-GFP became dispersed in wild-type pupal neurons (B), but remained punctate in mutant pupal neurons with *ik2-RNAi* (C), *ik2-G250D* (D) and *EcR-DN* (E) expression. (F) Quantitative analysis of the diameters and numbers of each Spn-F puncta in a single live ddaC neuron (the same neuron as in S1 Movie) at different time points. Each dot represents single Spn-F punctum in neuron at specific time point. (G) The average cytosolic fluorescent intensity of dispersed cytosolic GFP signals (an example indicated by asterisk in S2E Fig) was measured in the same ddaC neurons (n = 4 for each genotype) of larvae and at various time points of pupae in wild type and mutants with *ik2-RNAi*, *ik2-G250D* and *EcR-DN* expression. The fold changes were determined by dividing the average cytosolic fluorescent intensity of GFP signals in pupal ddaC neurons by that in the same larval cells, which is assigned as 1. (H-K) The activated Ik2 signals were detected by antibodies against phosphorylated Ik2 (P-Ik2) in larvae and pupae at 1 h APF. The strong P-Ik2 signals were observed in wild-type pupal C4da neurons at 1 h APF (I), but not in wild-type larval neurons (H), and not in mutant pupal neurons with either kinase-dead Ik2-G250D expression (J) or with dominantly-negative ecdysone receptor (*EcR-DN*) expression (K). (L) The average P-Ik2 signal intensity was measured in the cell body of ddaC neurons of wild-type larvae, and wild-type and mutant pupae at 1 h APF (n = 4 for each genotype). The fold changes were determined by dividing the average cytosolic fluorescent intensity of P-Ik2 signals in pupal ddaC neurons by that in larval cells, which is assigned as 1. Error bars show SD. Scale bars, 10 μm.

Given that the *spn-F-GFP* transgene could rescue observed defects in *spn-F* mutants, we concluded that this transgene could functionally substitute for the endogenous *spn-F* gene. Therefore, studying the role of Spn-F-GFP in C4da neurons should help us to uncover the molecular function of endogenous Spn-F in dendrite pruning. First, we examined the distribution of Spn-F-GFP in larval and pupal neurons. The Spn-F-GFP proteins displayed a punctate pattern in the soma, dendrites and axons of larval C4da neurons (Fig 3A and S2A Fig). However, those punctate Spn-F-GFP proteins were redistributed in the soma of pupal neurons at 5 h APF (Fig 3B). Since both Ik2 kinase and ecdysone are required for dendrite pruning [15], we asked whether Ik2 kinase activity and ecdysone signaling regulate the dispersion of Spn-F-GFP puncta in pupal neurons during pruning. To test this possibility, we examined the Spn-F-GFP distribution in mutant pupal neurons with expression of *ik2-RNAi*, kinase-dead mutant *ik2-G250D* [28] or dominantly negative ecdysone receptor (*EcR-DN*), and found that the Spn-F-GFP puncta remained intact in all three mutant neurons at 5 h APF (Fig 3C–3E). These results indicated that there is a negative correlation between Spn-F puncta and dendrite pruning, and both Ik2 kinase and ecdysone signaling are required to redistribute Spn-F from puncta in neurons during dendrite pruning. Moreover, we also found that redistribution of Spn-F from puncta was also observed in the larval C4da neurons with Ik2, but not with Ik2-G250D, overexpression (S2F–S2I Fig).

To gain mechanistic insight into the redistribution of Spn-F from puncta, we performed live-cell imaging to monitor Spn-F-GFP distribution in the same C4da neurons from larvae to pupae (S1 and S2 Movies). The live imaging and signal profiling of Spn-F-GFP showed high punctate and low dispersed signals in the cytosol of larval neurons (S2B and S2E Fig). We quantified the numbers and the sizes of Spn-F puncta in single live ddaC neurons, and found both numbers and sizes of Spn-F puncta decrease along the pupation time (Fig 3F). In contrast, the dispersed Spn-F-GFP signals in the cytosol of pupal neurons increased as the punctate signals decreased (S2C–S2E Fig). We quantified the averaged fluorescent intensity of dispersed cytosolic Spn-F-GFP signals in the soma, and measured the fold changes of these signals in the same neurons at various time points. In the cytosol of wild-type C4da neurons, the dispersed cytosolic GFP signals started to increase soon after pupation (Fig 3G), peaked at 1 h APF, and decreased to a steady state after 2 h APF. We noticed that these dispersed cytosolic signals measured in pupal neurons were all higher than those in larval cells (Fig 3G). Furthermore, this prompt increase of dispersed Spn-F-GFP signals in wild-type neurons was not observed in the mutant neurons with *ik2-RNAi*, *ik2-G250D*, or *EcR-DN* expression (Fig 3G), indicating that Ik2 kinase and ecdysone signaling promote the increase of dispersed Spn-F in early pupal neurons.

Next, we assessed Ik2 kinase activities in C4da neurons by staining with antibodies against phosphorylated Ik2 (P-Ik2) on serine 175, whose phosphorylation is essential for Ik2 activation [29]. Consistent with the observation of maximum dispersed Spn-F-GFP signals in the cell body of pupal neurons at 1 h APF (Fig 3G), strong P-Ik2 signals were detected in C4da neurons at the same stage (Fig 3I and 3L), whereas no specific signals were found in larval cells (Fig 3H and 3L) or in mutant neurons expressing Ik2-G250D (Fig 3J and 3L). These results indicated that Ik2 kinase activity is upregulated in early pupal neurons, but downregulated in larval neurons. Since transiently increasing Ik2 expression caused precocious dendrite severing of larval neurons (Fig 2B) [15], we concluded that elevation of Ik2 activity in pupal neurons initiates dendrite severing, and suppression of Ik2 activity in larval neurons prevents premature severing. We also examined P-Ik2 signals in mutant neurons with disrupted ecdysone signaling and failed to detect activated Ik2 signals (Fig 3K and 3L), indicating that Ik2 kinase acts downstream of ecdysone signaling in dendrite pruning. Taken together, these data demonstrate that Ik2 kinase is activated promptly in early pupal neurons and regulates Spn-F distribution in C4da neurons during dendrite pruning.

### Ik2-dependent phosphorylation of Spn-F is required for Spn-F distribution and dendrite pruning

**Fig 4:**
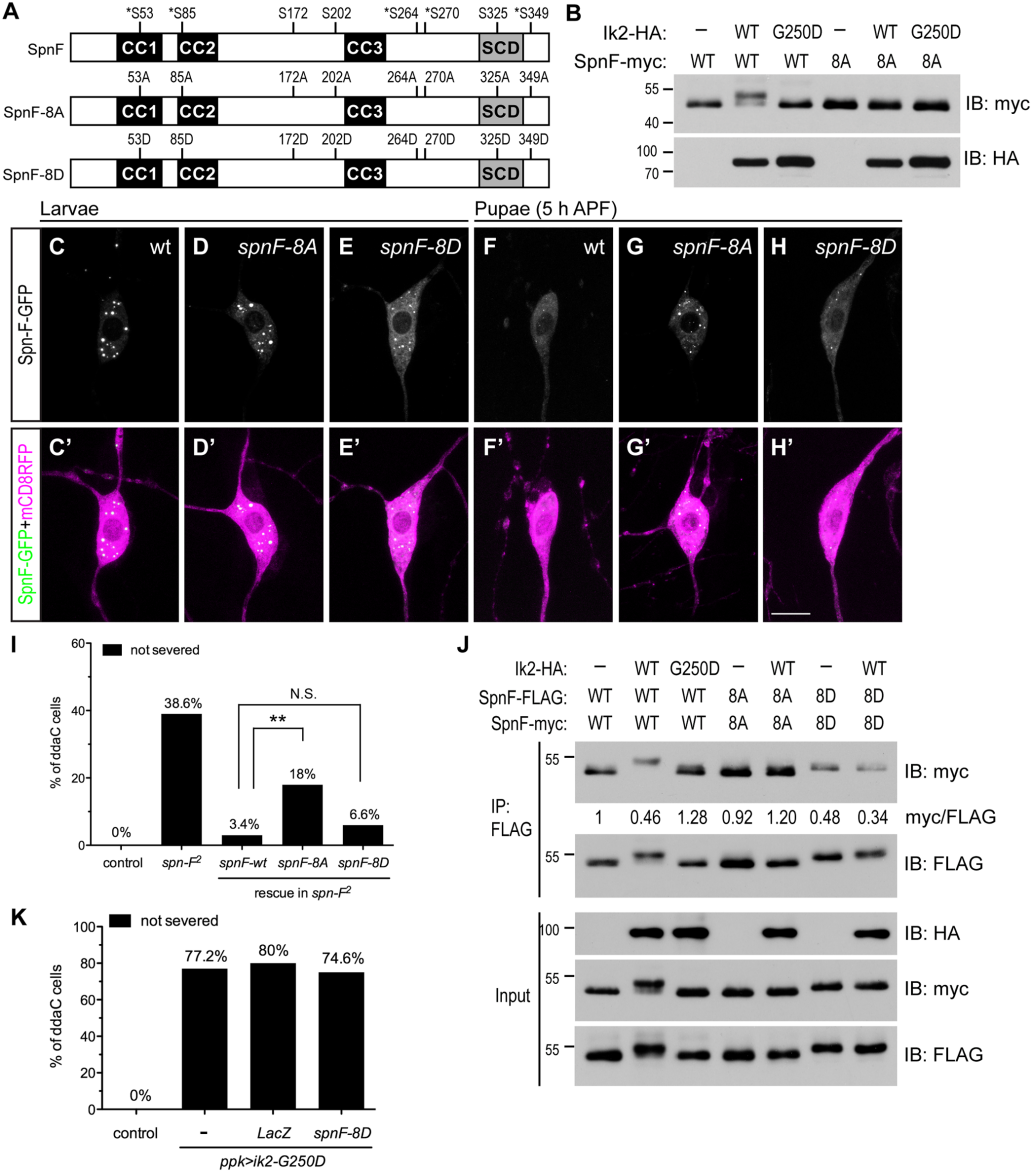
The phosphorylation of Spn-F by Ik2 kinase is required for Spn-F distribution and dendrite pruning. (A) Schematic shows the amino-acid residues of Spn-F protein phosphorylated by Ik2 kinase as identified by mass spectrometry. Five serine residues phosphorylated by Ik2 specifically are indicated by asterisks, and three serine residues with increased phosphorylation level in S2 cells with Ik2 overexpression are shown. These eight serine residues of Spn-F were mutated to alanines to generate phospho-resistant SpnF-8A mutant, or to aspartic acids to make phospho-mimetic SpnF-8D mutant. (B) Wild-type Spn-F or SpnF-8A was expressed in S2 cells alone, or together with wild-type Ik2 or kinase-dead Ik2-G250D mutant, and lysates were used for immunoblot analyses. (C'-H') The C4da neurons labeled by *ppk-GAL4* and *UAS-mCD8RFP* are shown. In ddaC neurons of wild-type larvae, SpnF-GFP (C), SpnF-8A-GFP (D) and SpnF-8D-GFP (E) showed punctate pattern. At 5 h APF, both SpnF-GFP (F) and SpnF-8D-GFP (H) puncta were dispersed, whereas SpnF-8A-GFP (G) remained as puncta. (I) Quantitative analysis of dendrite pruning phenotypes in ddaC cells at 16 h APF. SpnF-8D, not SpnF-8A, rescued pruning defects in ddaC neurons of *spn-F* ^*2*^ mutants. The percentage of ddaC neurons shows dendrite pruning defects among the total number of neurons examined. For wild-type control, n = 50; for *spn-F* ^*2*^ mutants, n = 70; for *spn-F* ^*2*^ mutants rescued with wild-type *spn-F*, n = 88; for *spn-F* ^*2*^ mutants rescued with *spnF-8A*, n = 100; for *spn-F* ^*2*^ mutants rescued with *spnF-8D*, n = 106. N.S., not significant. **, p = 0.0019. Statistical analysis was performed with Fisher’s exact test. (J) Co-IP experiments with lysates from S2 cells cotransfected with wild-type *spn-F*, *spnF-8A* or *spnF-8D* alone, and together with wild-type *ik2* or mutant *ik2-G250D* show that phosphorylation of Spn-F by Ik2 kinase decreases the self-association of Spn-F proteins. The myc/FLAG ratio (by ImageJ) indicates the relative amounts of Spn-F-myc protein associated with Spn-F-FLAG protein in co-IP experiments with anti-FLAG antibody. (K) Quantitative analysis of dendrite pruning phenotypes in ddaC cells at 16 h APF. The percentage of ddaC neurons shows dendrite pruning defects among the total number of neurons examined. For wild-type control, n = 100; for *ppk*>*Ik2-G250D* mutants, n = 92; for *ppk*>*Ik2-G250D*, *LacZ* mutants, n = 50; for *ppk*>*Ik2-G250D* mutants rescued with *spnF-8D*, n = 122.Scale bar, 10 μm.

The inverse correlation between Spn-F puncta and dendrite pruning suggests that redistribution of Spn-F from puncta in C4da neurons might be a critical event for dendrite pruning. If this is true, a mutant Spn-F that remains punctate and disperse-resistant in pupal neurons should have pruning defects. To generate such a mutant Spn-F, understanding the regulatory mechanism of Spn-F distribution is prerequisite. The detection of a band shifting of Spn-F-GFP, which is only expressed in C4da neurons, specifically in the larval lysates with Ik2 expression, but not with kinase dead Ik2-G250D expression or without Ik2 expression (S3 Fig), indicated that Spn-F could be phosphorylated by Ik2 kinase in C4da neurons. Since our study revealed that Ik2 kinase activity regulates Spn-F distribution, and Dubin-Bar *et al*. also shown that Ik2 phosphorylates Spn-F in kinase assays [26], we further characterized how the phosphorylation of Spn-F by Ik2 kinase regulates Spn-F distribution and dendrite pruning. First, to identify the residues of Spn-F phosphorylated by Ik2 kinase, we carried out mass spectrometry analyses. Given that Ik2-dependent redistribution of Spn-F-GFP in neurons could also be observed in *Drosophila* S2 cells, we reasoned that the mechanism underlying the reduction of Spn-F puncta is conserved in both cell types. Thus, we purified 1) Spn-F proteins from S2 cells with Ik2 expression referred to as the group of high Ik2 activity, and 2) Spn-F from S2 cells alone or 3) S2 cells with kinase-dead Ik2-K41A expression [30] referred as the group of low kinase activity for mass spectrometry analyses. The results revealed five serine residues (S53, S85, S264, S270 and S349) of Spn-F that are phosphorylated by Ik2 kinase specifically (S1 and S2 Tables and Fig 4A), and three other serine residues (S172, S202 and S325) showing increased level of phosphorylation in the group of high Ik2 activity (Fig 4A and S4 Fig). To determine the function of phospho-Spn-F, we substituted all eight serine residues with alanine to generate the phospho-deficient Spn-F-8A, and with aspartic acid to make the phospho-mimetic Spn-F-8D (Fig 4A). We observed evident gel mobility shift of Spn-F in the cell lysates of S2 cells co-expressing Ik2, but not Ik2-G250D (Fig 4B). In contrast, we detected no mobility shift of Spn-F-8A in any case (Fig 4B). This confirmed that the eight serine residues identified by mass spectrometry are the main sites of Spn-F phosphorylated by Ik2 kinase. Both Spn-F-8A and Spn-F-8D retained the interaction with Ik2 as the control Spn-F did (S5 Fig), consistent with previous reports that Ik2-dependent phosphorylation of Spn-F does not affect the interaction between Ik2 and Spn-F [26].

The presence of Spn-F puncta in cells suggested that Spn-F might form oligomers through self-association. To test this possibility, we performed co-immunoprecipitation (co-IP) in S2 cells and found that Spn-F proteins could interact with themselves (Fig 4J). Moreover, we observed reduced self-association of Spn-F in the S2 cells with Ik2 co-expression, but not in either the control S2 cells alone or the S2 cells with Ik2-G250D expression (Fig 4J). These results indicated that the phosphorylation of Spn-F by Ik2 kinase decreases Spn-F self-association. As expected, the self-association of Spn-F-8D, but not Spn-F-8A, was strongly reduced in both the control and the Ik2-expressing cells (Fig 4J). These data suggested a mechanism that Ik2 phosphorylates Spn-F to decrease Spn-F self-association and promotes the redistribution of Spn-F from punctate to dispersed in cytosol. Next, we examined the distribution of Spn-F-8A-GFP and -8D-GFP proteins in larval and pupal neurons respectively. The puncta formed by SpnF-8A-GFP were comparable to those formed by Spn-F-GFP in larval neurons (Fig 4C and 4D). However, the puncta formed by SpnF-8D-GFP were generally smaller than those by Spn-F-GFP in larval cells (Fig 4E). Moreover, the dispersed GFP signals in the cytosol of larval neurons with SpnF-8D-GFP expression were higher than those with Spn-F-GFP expression (Fig 4E). Notably, SpnF-8A-GFP puncta were resistant to disperse in neurons at 5 h APF (Fig 4G), while both Spn-F-GFP and SpnF-8D-GFP puncta were redistributed (Fig 4F and 4H). These findings confirmed that phosphorylation of Spn-F by Ik2 kinase promotes the dissociation of Spn-F oligomers and facilitates Spn-F redistribution in early pupal neurons.

Finally, to determine the role of Spn-F redistribution in dendrite pruning, we examined whether SpnF-8A, whose puncta are resistant to redistribution in pupal neurons, could rescue dendrite-pruning phenotypes in *spn-F* mutant neurons. Compared to the pruning defects rescued by wild-type Spn-F, we found Spn-F-8A is incapable of fully rescue the pruning defects in *spn-F* mutants (Fig 4I, p<0.005). This result confirmed that Spn-F redistribution in pupal C4da neurons is required for dendrite pruning. Moreover, SpnF-8D rescued the pruning phenotypes in neurons of *spn-F* mutants as efficiently as wild-type Spn-F did (Fig 4I). However, SpnF-8D cannot rescue the pruning defects in mutant neurons with *ik2-G250D* overexpression (Fig 4K), suggesting that other unidentified factors are required for dendrite pruning. Together, these results demonstrated that phosphorylation of Spn-F by Ik2 kinase, Spn-F redistribution and unidentified factors are critical for dendrite pruning in C4da neurons.

### Cytoplasmic dynein motor complex is required for Spn-F redistribution and dendrite pruning

**Fig 5:**
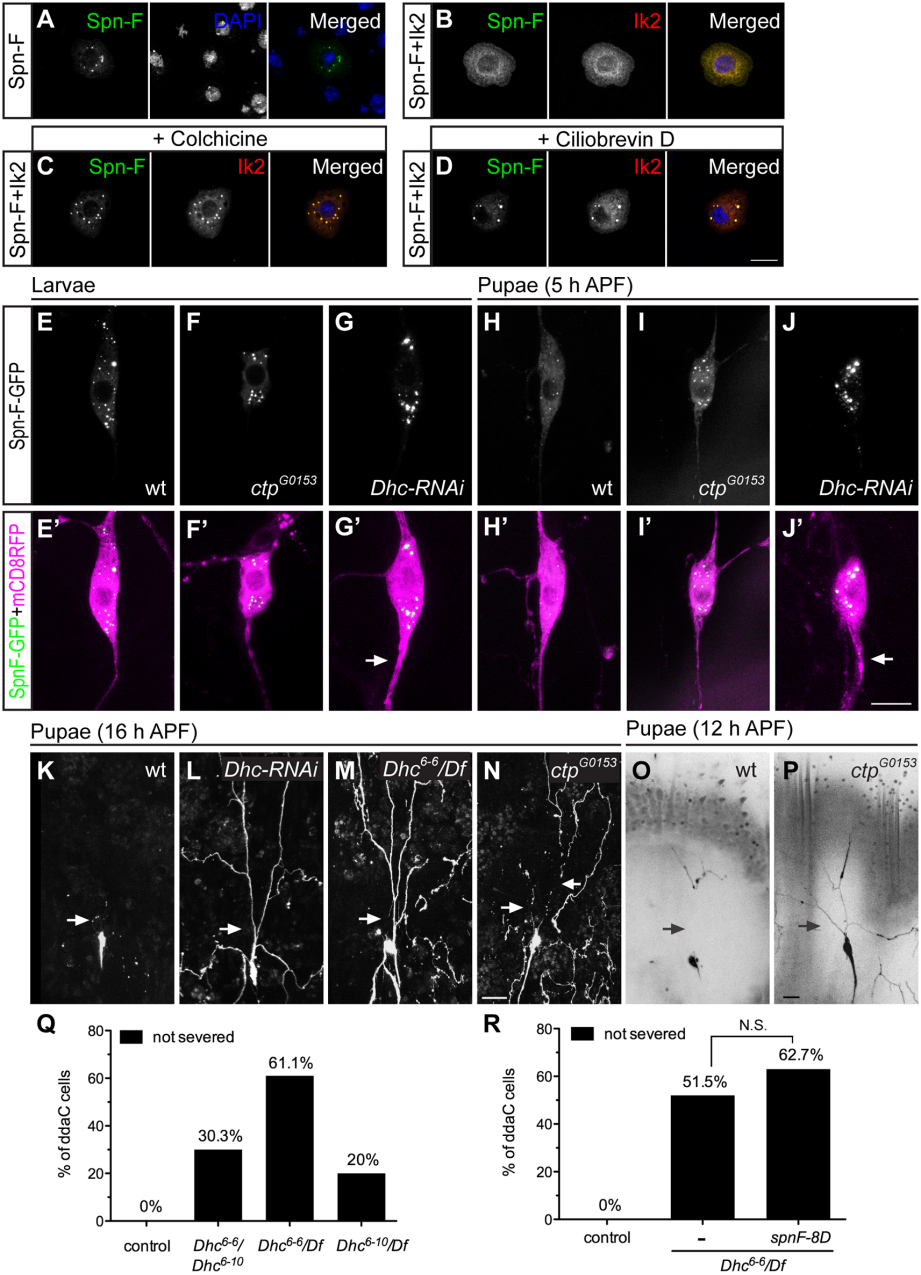
The function of cytoplasmic dynein complex is required for Spn-F redistribution and dendrite pruning. (A-D) Immunofluorescence of Spn-F-GFP, Ik2-HA and nuclear DAPI staining in S2 cells (A, B), in S2 cells treated with colchicine (C), and in S2 cells treated with ciliobrevin D (D). (E'-J') The ddaC neurons were visualized with *ppk-GAL4* and *UAS-mCD8RFP*. The Spn-F-GFP displayed as puncta in the soma of ddaC neurons of wild type (E), *ctp* ^*G0153*^ (F), and *Dhc-RNAi* (G) larvae. At 5 h APF, the Spn-F-GFP puncta were dispersed in ddaC neurons of wild-type pupae (H), but remained as puncta in both *ctp* ^*G0153*^ (I) and *Dhc-RNAi* (J) pupae. The thicken axons (arrows in G’ and J’) were caused by reduction of functional dynein complexes in *Dhc-RNAi* neurons. (K-P) The ddaC neurons were marked with *ppk-GAL4* and *UAS-mCD8GFP*. At 16 h APF, the dendrites of ddaC neurons were pruned in wild-type (K), but not in *Dhc-RNAi* (L), or *Dhc* ^*6-6*^ */Df* mutant (M) pupae. Df: deficiency line Df(3L)BSC436, which deletes *Dhc* gene. (N) The dendrites of ddaC cells were severed in the proximal dendrites (arrows), but not cleaned up in *ctp* ^*G0153*^ mutant pupae at 16 h APF. At 12 h APF, the dendrites of ddaC neurons are all severed (arrows) in wild-type pupae (O), but remained attached to the soma of ddaC cells in *ctp* ^*G0153*^ pupae (P). (Q) Quantitative analysis of dendrite pruning phenotypes in ddaC cells of *Dhc* mutants at 16 h APF. The percentage of ddaC neurons shows dendrite pruning defects among the total number of neurons examined. For wild-type control, n = 100; for *Dhc* ^*6-6*^ */Dhc* ^*6-10*^ mutants, n = 89; for *Dhc* ^*6-6*^ */Df* mutants, n = 90; for *Dhc* ^*6-10*^ */Df* mutants, n = 100. (R) Quantitative analysis of dendrite pruning phenotypes in ddaC cells of *Dhc* mutants with SpnF-8D expression at 16 h APF. The percentage of ddaC neurons shows dendrite pruning defects among the total number of neurons examined. For wild-type control, n = 100; for *Dhc* ^*6-6*^ */Df* mutants, n = 130; for *Dhc* ^*6-6*^ */Df* mutants rescued with *spnF-8D*, n = 110. Statistical analysis was performed with Chi-square test. N.S., not significant. Scale bars, 10 μm (A-J), 20 μm (K-P).

Since Spn-F redistribution from puncta is critical for dendrite pruning in C4da neurons, to further study the mechanism underlying Ik2-dependent Spn-F redistribution, we searched for other candidates that involve in Spn-F redistribution during dendrite pruning. Given that Spn-F interacts with a subunit of cytoplasmic dynein complex [24], Cut up (Ctp), the *Drosophila* homologue of dynein light chain 1 [31,32], and dynein is a microtubule-based motor protein, we questioned whether cytoplasmic dynein is involved in dendrite pruning. To address this question, we began to examine the roles of microtubules and cytoplasmic dyneins in Ik2-dependent redistribution of Spn-F in S2 cells with disrupted microtubule cytoskeletons or impaired dynein function. The redistribution of Spn-F from punctate to dispersed in the cytosol of S2 cells is also Ik2-dependent (Fig 5A and 5B). We found that Ik2-dependent Spn-F redistribution was suppressed in cells treated with microtubule-disrupting chemical, colchicine (Fig 5C). The similar observation was also obtained from S2 cells treated with cytoplasmic dynein specific inhibitor, ciliobrevin D [33] (Fig 5D). Notably, Spn-F puncta formed normally in S2 cells treated with either colchicine or ciliobrevin D (S6 Fig), suggesting that the intact microtubule cytoskeletons and functional dynein complexes are not required for the formation of Spn-F puncta. These results indicated that Ik2-dependent redistribution of Spn-F requires both functional dynein and intact microtubule cytoskeletons, and further suggested that Spn-F redistribution is caused by the movement of cytoplasmic dynein on microtubules in cells.

Next, to verify whether the function of cytoplasmic dynein complexes is required to redistribute Spn-F in neurons for dendrite pruning, we examined the distribution of Spn-F-GFP in mutant C4da neurons with impaired dynein function. Considering the interaction between Spn-F and Ctp proteins, we first examined the Spn-F-GFP distribution in *ctp* mutant neurons. Since *ctp* is an essential gene for embryonic development [32], we used a hypomorphic *ctp* mutant allele *ctp* ^*G0153*^, which had few mutant larvae developing to pupae, for analyses. The Spn-F-GFP puncta formed normally in larval neurons of *ctp* ^*G0153*^ mutants (Fig 5F, compared to Fig 5E), but failed to disperse in mutant pupal neurons at 5 h APF (Fig 5I, compared to Fig 5H). We next examined the role of *dynein heavy chain* (*Dhc*), which encodes the only motor subunit in dynein complex, in the redistribution of Spn-F in C4da neurons. Among seven *Dhc* genes in fly genomes [34], *Dhc64C* (thereafter referred to as *Dhc*) is the only one exhibiting ubiquitous expression, including the nervous systems, throughout development [35]. We employed *Dhc* RNAi lines to knockdown endogenous Dhc. The thick axons found in larval neurons with *Dhc* dsRNAs expression (Fig 5G’, compared to Fig 5E’) were consistent with reported phenotypes in mutant neurons with impaired dynein function [36], and validated the knockdown efficiency. The Spn-F-GFP puncta formed normally in larval neurons of *Dhc-RNAi* mutants (Fig 5G), but remained intact in mutant neurons at 5 h APF (Fig 5J). These findings demonstrated the requirement of functional dynein complex to redistribute Spn-F in C4da neurons during dendrite pruning.

Finally, to verify the roles of dynein complexes in dendrite pruning, we examined whether mutant C4da neurons with impaired dynein function show pruning defects. At 16 h APF, the primary dendrites remained connected to the soma of C4da neurons in *Dhc* RNAi mutants (Fig 5L, compared to Fig 5K), suggesting a critical role of dynein motor in dendrite pruning. We also verified the dendrite pruning defects in neurons of trans-heterozygous *Dhc* mutants with hypomorphic alleles, *Dhc* ^*6-6*^ and *Dhc* ^*6-10*^ (Fig 5Q) [37]. In order to confirm that the phenotypes observed in hypomorphic *Dhc* mutants were not due to other unidentified mutations on the same chromosome, we examined C4da neurons of *Dhc/Df* mutants (deficiency uncovers the entire *Dhc64C* gene). The defective dendrite pruning was evident in C4da neurons of *Dhc/Df* mutants; 61% (n = 90) of ddaC neurons in *Dhc* ^*6-6*^ */Df* mutants (Fig 5M and 5Q) and 20% (n = 100) of cells in *Dhc* ^*6-10*^ */Df* mutants (Fig 5Q) showed phenotypes at 16 h APF. Since dynein has been shown to play an essential role in larval dendrite development [36], to verify the pruning defects in *Dhc* mutants were not secondary to the early dendrite development defects, we examined the dendritic morphology of C4da neurons in *Dhc* mutant larvae, and observed normal dendrites of most larval C4da neurons in *Dhc* mutants (S7 Fig). These findings demonstrated that *Dhc* plays a critical role in dendrite pruning of C4da neurons. Furthermore, at 16 h APF, about 22.5% of C4da neurons (n = 80) in hypomorphic *ctp* ^*G0153*^ mutants exhibited severe dendrite clearance phenotypes, which is characterized by long disconnected dendrites surrounding the soma (Fig 5N). We hypothesized that *ctp* ^*G0153*^ mutant neurons might have delayed dendrite severing, which resulted in deferred clearance. To test this hypothesis, we examined the dendrites of C4da neurons in wild-type and *ctp* ^*G0153*^ mutant pupae at 12 h APF. All dendrites of wild-type neurons (n = 38) were severed at 12 h APF (Fig 5O); however, about 26.8% of neurons (n = 56) in *ctp* ^*G0153*^ mutants still retained their primary dendrites attached to the soma at the same stage (Fig 5P). These results indicated that dendrite severing was delayed in hypomorphic *ctp* mutant neurons, and confirmed that *ctp* plays a role in dendrite pruning. Finally, we asked whether SpnF-8D could rescue the dendrite pruning defects observed in *Dhc* mutants. We found no significant difference between the pruning defects of C4da neurons in *Dhc* ^*6-6*^ */Df* mutants and that in *Dhc* mutants with SpnF-8D overexpression at 16 h APF (Fig 5R), indicating that SpnF-8D cannot rescue the dendrite pruning defects in *Dhc* mutants. Taken together, our data demonstrates that cytoplasmic dynein motor complex is required for Spn-F redistribution in C4da neurons and for dendrite pruning.

### Spn-F links Ik2 kinase to cytoplasmic dynein motor complex

**Fig 6:**
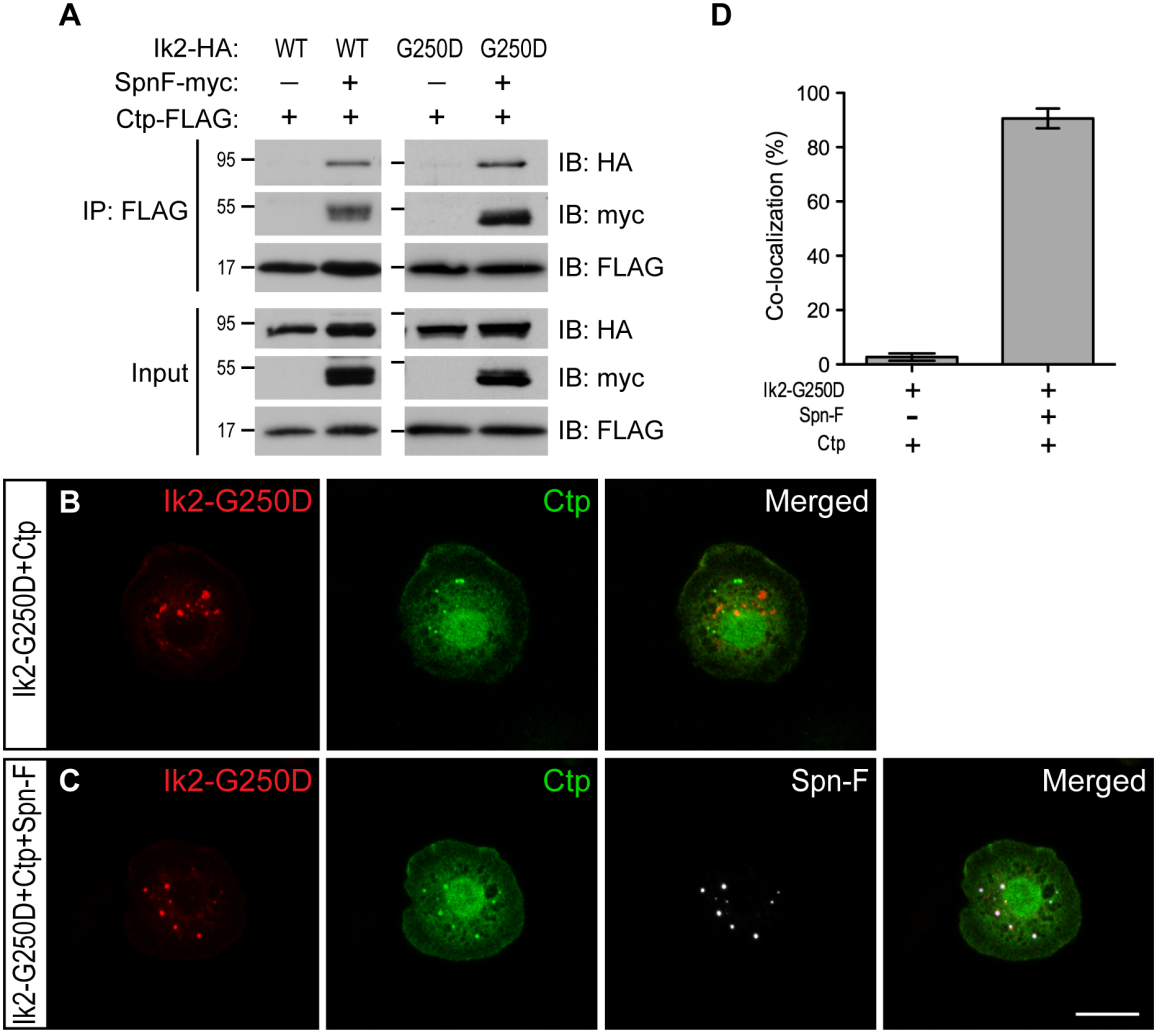
Spn-F links Ik2 kinase to dynein complexes via SpnF/Ctp interaction. (A) Co-IP assays were performed with lysates from S2 cells transfected with Spn-F-myc, Ctp-FLAG and Ik2-HA or Ik2-G250D-HA to show that the formation of Ik2 (or Ik2-G250D)/Spn-F/Ctp tertiary complexes depends on the presence of Spn-F proteins. (B) Immunofluorescent staining in S2 cells transfected with Ik2-G250D-HA and Ctp-FLAG showed no colocalization of Ik2-G250D and Ctp. (C) Immunofluorescent staining in S2 cells transfected with Ik2-G250D-HA, Spn-F-GFP and Ctp-FLAG showed colocalization of Ik2-G250D, Spn-F and Ctp. (D) Quantitative analysis of colocalization of Ik2-G250D/Ctp and Ik2-G250D/Spn-F/Ctp in transfected S2 cells. The percentage of S2 cells shows colocalization of staining signals among the total number of examined cells that expressed all transfected proteins. The percentages of colocalization were calculated from three independent experiments: for Ik2-G250D/Ctp transfected cells, n = 5/116, 2/102 and 2/105; for Ik2-G250D/Spn-F/Ctp transfected cells, n = 102/111, 100/107 and 96/111. Error bars show SD. Scale bar, 10 μm.

Since Spn-F interacts with both Ik2 and Ctp [24,26], we hypothesized that Ik2, Spn-F and Ctp might form ternary complexes, which could be transported by cytoplasmic dynein. To test this hypothesis, we performed co-IP and antibody staining in S2 cells. The formation of Ik2/Spn-F/Ctp ternary complexes could be detected by co-IP only from the lysates of S2 cells with Spn-F co-expression, but not from that without Spn-F co-expression (Fig 6A), suggesting the central role of Spn-F in the ternary complex formation. As Ik2 kinase causes Spn-F redistribution, the even distribution of all three molecules observed in S2 cells (S8 Fig) did not provide convincing evidence to support the colocalization. To visualize the colocalization of these three molecules in S2 cells, we used Ik2-G250D for antibody staining based on the following reasons. First, the dependence of Spn-F to form ternary complex was also observed between Ik2-G250D and Ctp proteins (Fig 6A). Second, since Ik2 kinase activity does not affect Ik2/Spn-F interaction [26], kinase-dead Ik2-G250D still possesses normal interaction with Spn-F and remains as puncta in S2 cells. The immunofluorescence staining (Fig 6B and 6C) and quantification (Fig 6D) showed that the colocalization of Ik2-G250D, Spn-F and Ctp molecules were observed only in S2 cells with Spn-F co-expression, but not in cells without Spn-F co-expression, supporting the formation of Ik2/Spn-F/Ctp ternary complex in cells. To test whether Spn-F phosphorylation by Ik2 kinase may affect the formation of Ik2/Spn-F/Ctp complexes, we performed co-IP and colocalization experiments with SpnF-8A and SpnF-8D in S2 cells. Our results showed that SpnF-8A and SpnF-8D have similar abilities as wild-type Spn-F in mediating the formation of Ik2/Spn-F/Ctp complexes in S2 cells (S9 Fig), suggesting that Spn-F phosphorylation have no or minor effects on Ik2/Spn-F/Ctp complex formation. These data demonstrated that Spn-F acts as a central mediator to link Ik2 kinase to dynein motor complex via Spn-F/Ctp interaction in cells.

### The Ik2/Spn-F/Ctp complex is critical for Spn-F redistribution and dendrite pruning

**Fig 7:**
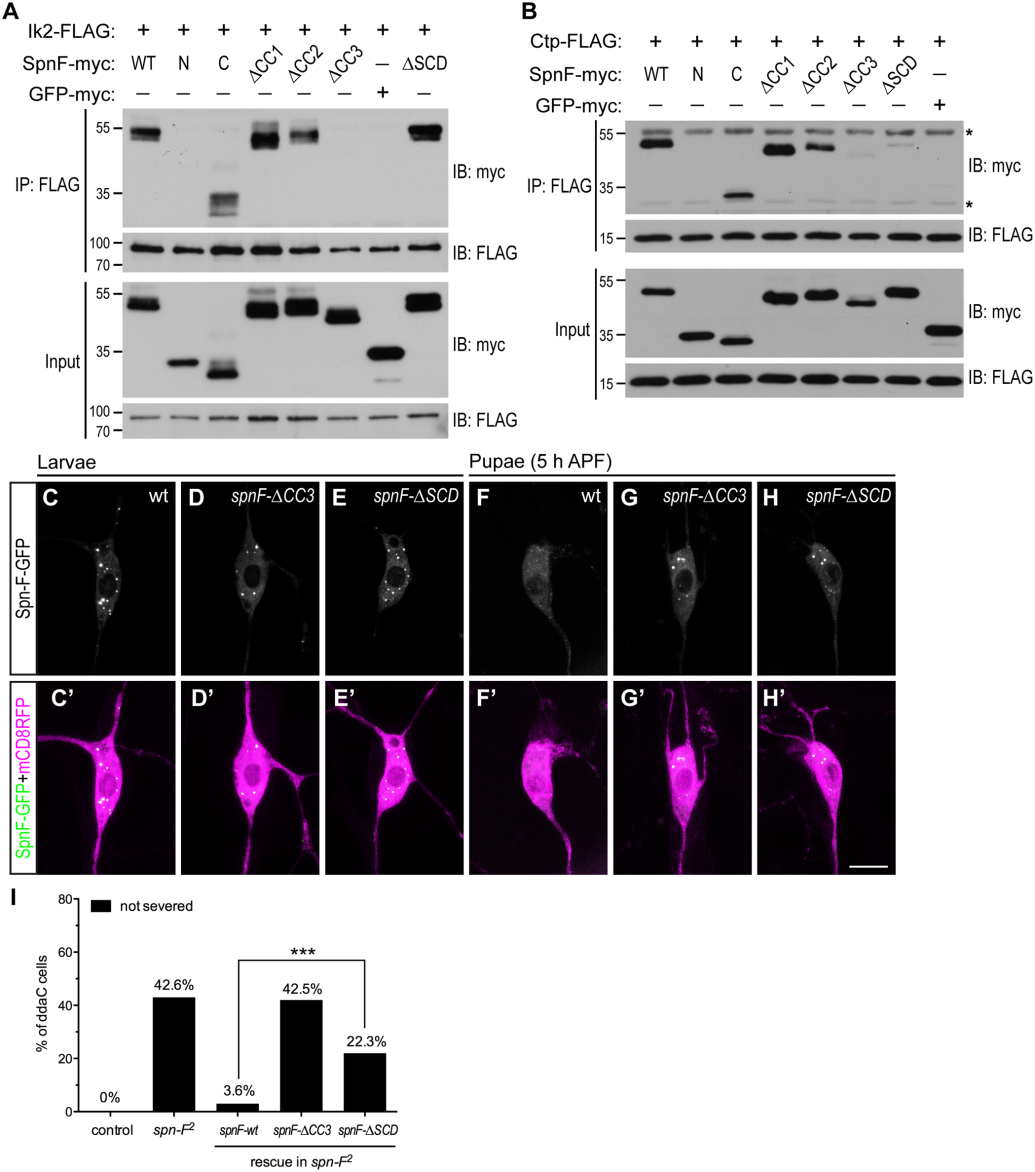
The formation of Ik2/Spn-F/Ctp complex is critical for Spn-F redistribution and dendrite pruning. (A) The CC3 domain of Spn-F is the Ik2-interacting domain. Ik2-FLAG and full-length or Spn-F mutants lacking designated domain were expressed in S2 cells and used to make lysates for co-IP assays. GFP-myc was used as a negative control. (B) Both CC3 and SCD domains of Spn-F are required for Ctp binding. Ctp-FLAG and full-length or truncated Spn-F were expressed in S2 cells and lysates were prepared for co-IP with anti-FLAG antibodies. Asterisks indicate the position of heavy and light chains of antibodies. (C'-H') The ddaC neurons were visualized with *ppk-GAL4* and *UAS-mCD8RFP*. The Spn-F-GFP exhibited puncta in the soma of ddaC neurons at larval stages (C), but dispersed in the pupal neurons at 5 h APF (F). The mutant SpnF-ΔCC3-GFP showed puncta in larval ddaC neurons (D), but remained as puncta in pupal neurons at 5 h APF (G). The mutant SpnF-ΔSCD-GFP formed puncta in larval ddaC neurons (E), and remained as puncta in pupal neurons at 5 h APF (H). (I) Quantitative analysis of pruning defects in ddaC neurons at 16 h APF. The percentage of ddaC neurons shows pruning defects among the total number of neurons examined. For wild-type control, n = 50; for *spn-F* ^*2*^ mutants, n = 110; for *spn-F* ^*2*^ mutants rescued with wild-type *spn-F*, n = 88; for *spn-F* ^*2*^ mutants rescued with *spnF-ΔCC3*, n = 73; for *spn-F* ^*2*^ mutants rescued with *spnF-ΔSCD*, n = 121. ***, p<0.0001. Statistical analysis was performed with Fisher’s exact test. Scale bar, 10 μm.

As Ik2, Spn-F and Ctp are crucial for dendrite pruning, and Spn-F plays a central role in the formation of Ik2/Spn-F/Ctp ternary complex in cells, we asked whether this ternary complex is required for the Spn-F redistribution and for dendrite pruning in C4da neurons. To approach this question, we set out to map the Ik2- and Ctp-interacting domains of Spn-F, and determined the role of Ik2/Spn-F/Ctp ternary complex in neurons for dendrite pruning. Spn-F protein contains three coiled-coil domains [38], namely CC1, CC2 and CC3, and an Spn-F conserved domain (SCD) at its C terminus (Fig 4A), which is evolutionarily conserved among different insect species (S10A Fig). To identify the Ik2- and Ctp-interacting domains of Spn-F, we generated a series of Spn-F deletion mutants (S10B Fig) and examined their interactions with Ik2 and Ctp in S2 cells by co-IP. The results showed that removal of Spn-F CC3 domain completely abolished the interaction between Ik2 and Spn-F (Fig 7A), but remained weak interaction between Spn-F and Ctp (Fig 7B), indicating that both Ik2/Spn-F and Spn-F/Ctp interactions are disrupted in SpnF-ΔCC3. Moreover, the co-IP experiments revealed normal interaction between Ik2 and SpnF-ΔSCD (Fig 7A), but weak interaction between SpnF-ΔSCD and Ctp (Fig 7B), demonstrating that SpnF-ΔSCD keeps a normal interaction with Ik2, but a defective interaction with Ctp. Both the SpnF-ΔCC3 and -ΔSCD puncta formed normally in larval neurons (Fig 7D and 7E), comparable to the wild-type Spn-F puncta (Fig 7C); however, both Spn-F deletion mutants remained punctate in neurons at 5 h APF (Fig 7G and 7H, compared to Fig 7F), indicating that both Spn-F CC3 and SCD domains are crucial for Spn-F redistribution in pupal neurons. Finally, to determine the role of Ik2/Spn-F/Ctp ternary complex in C4da neurons for dendrite pruning, we performed rescue experiments with SpnF-ΔCC3-GFP and -ΔSCD-GFP in *spn-F* mutant neurons. SpnF-ΔCC3-GFP failed to rescue the dendrite pruning defects of ddaC neurons in *spn-F* mutants at 16 h APF (Fig 7I), demonstrating that the Spn-F CC3 domain, which mediates both Ik2/Spn-F and Spn-F/Ctp interactions, is essential for dendrite pruning in C4da neurons. In contrast to SpnF-ΔCC3-GFP, SpnF-ΔSCD-GFP only partially rescued the dendrite pruning defects in *spn-F* mutants at 16 h APF (Fig 7I), suggesting the role of Spn-F/Ctp interaction in facilitating dendrite pruning. Collectively, our data demonstrated that the Ik2/Spn-F/Ctp complex is critical for Spn-F redistribution and for dendrite pruning in C4da neurons.

## Discussion

In addition to apoptosis, neurons have a second self-destruct program in their axons for axonal pruning during development and in response to neuronal injury and disorders [39]. Here, we propose a third self-destruct program, which is mediated by Ik2 kinase activity in *Drosophila* sensory neurons, specific for dendrite pruning. Ik2 is essential for dendrite severing in pupal C4da neurons [15], and currently is the only known molecule sufficient to cause precocious dendrite severing in larval cells [15], indicating that Ik2 activation must be regulated temporally. For temporal regulation, ecdysone signaling plays a key role in dendrite pruning [13,14]. Our studies showing that no Ik2 activation was detected in pupal C4da neurons with impaired ecdysone signaling and thus placed Ik2 kinase downstream of ecdysone signaling. Microarray studies have identified *ik2* as one of the ecdysone/EcR up-regulated genes in brain MB γ neurons during axon pruning [40]. This suggests one possible mechanism where ecdysone/EcR regulates Ik2 activation through increasing *ik2* expression in C4da neurons. Although Ik2 kinase activity is crucial for oogenesis and bristle morphogenesis [25,29], the activation mechanisms of Ik2 kinase in both processes remain unknown. Since pruning activity is considered as a self-destruct program, how to regulate this activity spatially in subcellular compartments within individual neurons is an intriguing issue to investigate. In this study, we identified Spn-F and cytoplasmic dynein complex as critical regulators of Ik2-mediated dendrite pruning activity in C4da neurons.

It is known that endogenous Spn-F exhibits a punctate pattern in nurse cells [24], consistent with our observation of punctate Spn-F-GFP in larval C4da neurons. The formation of Spn-F puncta in cells is through self-association, and does not depend on the integrity of microtubule network or the function of cytoplasmic dynein (S6 Fig). Since Ik2 could form oligomers in cells [29], the interaction between Ik2 and Spn-F might also play a role in Spn-F puncta formation. Indeed, we observed that SpnF-ΔCC3-GFP has normal interaction with either SpnF-ΔCC3 or full-length Spn-F (S11 Fig), but formed fewer puncta than the wild type Spn-F-GFP did in larval neurons (Fig 7D). Therefore, the Spn-F puncta formation could be attributed not only to Spn-F self-association, but also to Ik2/Spn-F interaction and Ik2 oligomerization.

In larval C4da neurons, Ik2 kinase is inactive and associates with Spn-F as puncta in the cytosol. After puparium formation, Ik2 kinase becomes activated promptly and phosphorylates Spn-F in C4da neurons. This Ik2-dependent phosphorylation on Spn-F decreases Spn-F self-association, and subsequently the numbers and sizes of Spn-F puncta were reduced (Fig 3F). One may question that protein degradation might contribute to decrease the numbers and sizes of Spn-F puncta in C4da neurons during dendrite pruning. It was known that Ik2 promotes caspase inhibitor DIAP1 degradation via proteasomes during the development of sensory organ precursors [28]; therefore, Ik2 might promote Spn-F degradation in C4da neurons during dendrite pruning. However, Ik2 overexpression does not alter the protein level of Spn-F in either S2 or germline cells [26]. Thus, protein degradation by proteasomes is unlikely the mechanism leading to decreased Spn-F puncta after Ik2 activation. Since P-Ik2 signals were indistinguishable between wild-type and *Dhc64C* RNAi neurons (S12 Fig), it is reasonable to presume that both Ik2 activation and Spn-F phosphorylation occur normally in dynein mutant neurons. We also found no significant differences between the pruning defects of C4da neurons in *spn-F* mutants and that in *spn-F* mutants with *Dhc-RNAi* (S12D Fig), and between the pruning phenotypes observed in *Dhc* mutants and that in *Dhc* mutants with *ik2-RNAi* (S12E Fig). These findings further support that Ik2, Spn-F and dynein complex function together in the same pathway in dendrite pruning of C4da neurons. However, our finding of Spn-F puncta in mutant pupal neurons with impaired dynein function indicated that dynein is required for Spn-F redistribution after Ik2 activation. Furthermore, Spn-F remains punctate in S2 cells with Ik2 overexpression even after microtubule depolymerization and inhibition of dynein function, suggesting that dynein might redistribute Ik2/Spn-F complexes via transporting complexes toward the minus ends of microtubules in C4da neurons during dendrite pruning. Based on our results in this study and studies in germline cells that more Spn-F puncta accumulated in nurse cells with colchicine treatment and with *Dhc* mutation [24], we favored the mechanism of protein redistribution for Spn-F punctum reduction in dendrite pruning of C4da neurons.

It has been shown that during *Drosophila* bristle elongation, directional transport of activated Ik2 and of Spn-F to the bristle tips, where the microtubule minus ends are concentrated [41], requires the function of cytoplasmic dynein, and Spn-F acts as an adaptor to link Ik2 to dynein complexes [42]. These are similar to our findings that both Ik2 activation and dynein complex are essential for Spn-F redistribution, and Spn-F plays a central role in the formation of Ik2/Spn-F/dynein complex, which is crucial for Spn-F redistribution and for dendrite pruning in C4da neurons. However, the studies in bristle elongation indicating that *spn-F* acts upstream of *ik2* [42] disagree with our finding that *ik2* acts upstream of *spn-F* in dendrite pruning. The discrepancy between the epistasis analyses of *ik2* and *spn-F* in these two different processes might be due to different cell-type specific factors in these two types of cells that affect the morphological readouts in genetic studies. Moreover, we demonstrated that Ik2-dependent phosphorylation of Spn-F decreases Spn-F self-association, promotes Spn-F redistribution, and finally leads to dendrite pruning in C4da neurons.

The activated Ik2 signals are found accumulated at the microtubule minus ends in cells with polarized microtubule distribution, such as oocytes, follicle cells and bristles [29,43]. This is consistent with our conclusion that dynein transports activated Ik2 toward microtubule minus ends in C4da neurons. Since *Drosophila* sensory neurons have polarized microtubules in their proximal dendrites predominantly with microtubule minus end pointing away from the cell body [18], our studies revealed a possible mechanism that Spn-F and minus-end directed motor dynein complex confine Ik2-dependent pruning activity to the somatodendritic compartments of C4da neurons. During *Drosophila* bristle elongation, the accumulation of endogenous Spn-F observed at the bristle tip, where the microtubule minus ends are enriched [41], led us to examine Spn-F-GFP signals along the dendrites of C4da neurons during dendrite pruning. However, we did not observe enriched Spn-F-GFP signals in the proximal dendrites, where dendrite severing is expected to occur, by live imaging during pruning. Previous studies [15] showed that microtubules are first disassembled in the proximal dendrites of C4da neurons during dendrite severing, and this local disassembly of microtubules was suppressed in *ik2* mutant neurons. Since our genetic studies indicated that both *ik2* and *spn-F* acts in the same pathway of dendrite pruning, we tested whether local microtubule disassembly happens normally in *spn-F* mutants. We found that local breakage of microtubules in the proximal dendrites of C4da neurons was also suppressed in *spn-F* RNAi mutants (S13 Fig), suggesting that Spn-F, like Ik2, plays a role in dendrite severing that involves local microtubule disassembly. However, the molecular mechanisms by which activated Ik2 and Spn-F lead to dendrite severing in the proximal dendrites of C4da neurons will be an important question for future studies.

It is known that there was no decrease in cell death in wing discs with *ik2* knockdown and in *ik2* mutant embryos [28], indicating that the primary function of Ik2 is not involved in apoptotic pathway during development. However, ectopic Ik2 activation by overexpression leads to cell death in fly compound eyes [28] and in C4da neurons [15], suggesting that excessive Ik2 kinase signaling could trigger a crosstalk with signaling molecules in apoptotic pathway and result in apoptosis. It is known that Ik2 kinase regulates the nonapoptotic function of caspase through promoting DIAP1 degradation [28]. In a similar manner, the confinement of activated Ik2 kinase in the dendritic compartments might restrict the detected caspase activity in the degenerating dendrites after separating from the soma of C4da neurons during dendrite pruning [21]. Therefore, this raises a possibility that de-regulation of pruning activity in neurons may trigger a crosstalk with molecules in apoptotic pathway and lead to undesired cell death during neuronal injury and disorders. Recently, a caspase cascade, including caspase 3 and 6, was identified in mice to play a role in developmental axon pruning and in sensory axon pruning after trophic factor withdrawal [44,45]. Moreover, activated caspase 6 was detected in human patient brains of Alzheimer and Huntington diseases long before cell death [46], highlighting a critical role in regulating caspase activity in both diseases. Understanding the regulatory mechanisms that confine pruning activity into proper subcellular compartments of the neuron might provide molecular insights into the pathogenesis of neural disorders.

## Materials and Methods

### Fly stocks

*spn-F* ^*2*^ [24], *Dhc* ^*6-6*^, *Dhc* ^*6-10*^ [37], *ppk-eGFP* [47], *ppk-GAL4* [27], *UAS*-*ik2* (*UAS*-*DmIKK ε*), and *UAS*-*ik2*-*G250D* [28] have been described previously. *Df(3R)tll-e*, *Df(3L)BSC436*, *tub-GAL80* ^*ts*^, and *UAS-EcR-F645A* were obtained from Bloomington Stock Center. *ctp* ^*G0153*^ was obtained from *Drosophila* Genetic Resource Center (DGRC). RNAi lines were obtained from Vienna *Drosophila* RNAi Center (VDRC), and Fly Stocks of National Institute of Genetics (NIG-FLY). Flies were raised at 25°C. For the experiments with *tub*-*GAL80* ^*ts*^, flies were transferred and kept at 29°C for 40–42 h before analysis and imaging. Transgenic flies were generated by standard P-element mediated transformation.

### Evaluation and quantification of the pruning phenotype

Our previous study with ddaC neurons labeled with *ppk-eGFP* or *ppk-GAL4*::*UAS-mCD8GFP* indicated normal severing occurs around 4–6 h APF, and complete debris clearance around 16–18 APF [15]. Therefore, we analyzed the pruning phenotype of ddaC neurons at or after 16 h APF to exclude individual temporal differences. At time of analysis, ddaC neurons having any single dendrite branch with continuous GFP signals extended from the center of soma more than 100 μm was considered pruning defective.

### Molecular cloning

The *spn-F* full-length and deletion DNA fragments were generated by using PCR to amplify the DNA fragments from the cDNA clone LD01470 (Drosophila Genomics Resource Center) as the template. The *spnF-8A* and *spnF-8D* DNA fragments were generated by PCR-based site-directed mutagenesis. All *spn-F* DNA fragments were inserted together with EGFP coding sequence or the coding sequences containing HA, myc, or FLAG tags into *pUAST* vector to make various *UAS-spn-F* constructs. To generate *UAS-ctp-FLAG* construct, a DNA fragment encoding Ctp protein was amplified by PCR from the genomic DNA of *w* ^*1118*^ flies and inserted together with FLAG coding sequence into *pUAST* vector. The DNA sequences of all *spn-F* and *ctp* inserted fragments were verified by DNA sequencing.

### Cell culture, immunoblot and co-immunoprecipitation assays

*Drosophila* Schneider cells (S2 cells) were cultured in Shields and Sang M3 insect medium (Sigma) supplemented with 10% FCS and antibiotics at 25°C. Plasmids were transfected using Effectene (QIAGEN). To disrupt microtubule cytoskeletons, S2 cells were treated with 10 μM cholchicine. To suppress dynein function, S2 cells were treated with 10 μM ciliobrevin D. Cells were transfected with *tubulin-GAL4* to drive various UAS constructs: *UAS-ik2-HA*, *UAS-ik2-FLAG*, *UAS-ik2-G250D-HA*, *UAS-spn-F-myc*, *UAS-spn-F-FLAG*, *UAS-spnF-8A-myc*, *UAS-spnF-8D-myc*, *UAS-spnF-N-myc*, *UAS-spnF-C-myc*, *UAS-spnF-ΔCC1-myc*, *UAS-spnF-ΔCC2-myc*, *UAS-spnF-ΔCC3-myc*, *UAS-spnF-ΔSCD-myc*, *UAS-GFP-myc*, and *UAS-ctp-FLAG*. Cells were lysed in lysis buffer (20 mM Tris pH 7.0, 150 mM NaCl, 2 mM EDTA, 1% TritonX-100, 1 mM DTT and protease inhibitors (Roche)). The cell lysates were incubated with anti-FLAG M2 agarose beads (Sigma) on ice and washed thoroughly with lysis buffer. Proteins were eluted and detected using immunoblot assays with rat antibody to HA (Roche), rabbit antibody to Myc (Santa Cruz Biotechnology), mouse antibody to FLAG (Sigma), and appropriate HRP-conjugated secondary antibodies (Jackson Immuno Research).

### Immunohistochemistry and live imaging

Primary antibodies used in this study were rabbit anti-P-Ik2 antibodies (1:50, a gift of Dr. S. Hayashi) [29], rat anti-HA 3F10 (1:500, Roche), mouse anti-FLAG (1:500, Sigma). The secondary antibodies conjugated with DyLight dyes (Jackson Immuno Research) were used at 1:500 dilution. The larvae and pupae were dissected in cold PBS, fixed with 4% formaldehyde for 20 min at room temperature, and stained with proper primary antibodies and subsequent secondary antibodies. For antibody staining in S2 cells, transfected S2 cells were transferred to coverslips coated with Concanavalin A (Sigma), fixed with 4% formaldehyde, and stained with proper primary antibodies and appropriate secondary antibodies. For live imaging, pupae were placed on a double-sided tape (3M) with dorsal side up, and pupal cases were removed by forceps. The pupae were overlaid with distilled water and coverslip for imaging using laser scanning confocal microscope. Images of fluorescent signals were acquired in live animals or fixed samples on confocal microscopes of Bio-Rad MRC-600, Leica TCS SP5, or Zeiss LSM 710. The z-stack images were collected and the maximum intensity projection was used for further analysis. The images were processed using ImageJ (National Institutes of Health), and brightness and contrast were adjusted by Photoshop (Adobe).

### Timelapse imaging and quantification

For timelapse imaging of Spn-F-GFP distribution in ddaC neurons from larvae to early pupae, larvae and pupae were placed with dorsal side up, overlaid with halocarbon oil and coverslip, and imaged with Zeiss LSM 510 laser scanning confocal microscope. To analyze the cytosolic fluorescence of Spn-F-GFP in neurons, we first drew a line across the center of nucleus of cells, and applied the profile analysis module of the Zen software (Zeiss) to reveal the signal intensity along the line (an example in S1B and S1E Fig). The peaks of fluorescence intensity were considered as Spn-F-GFP puncta, and the rest of cytosolic fluorescence, which excludes the peaks, was considered as the dispersed cytosolic Spn-F-GFP in cells (an example as a region marked by an asterisk in S1E Fig). The cumulative fluorescence intensities of dispersed cytosolic Spn-F-GFP (excluding the signals of any Spn-F-GFP puncta) were calculated and divided by the pixels numbers to obtain the average dispersed fluorescence intensity in the cytosol of each neuron. The average cytosolic dispersed fluorescence intensity of larval neurons was considered as one to calculate the fold changes of average cytosolic dispersed signals in the same neuron at various time points after pupation. Statistical analysis was conducted and graphs were generated in GraphPad Prism.

### NanoLC-MS/MS analysis of phosphorylated Spn-F

Spn-F proteins were isolated by anti-FLAG M2 beads (Sigma) from S2 cells transfected with *tubulin-GAL4* and *UAS-spn-F-FLAG* alone, or together with either *UAS-ik2* or *UAS-ik2-K41A*. The bound proteins were eluted with FLAG peptide (Sigma), separated by SDS-PAGE, and stained with Coomassie brilliant blue R-250 (Sigma). The Spn-F-FLAG protein bands were manually excised from the gel and performed in-gel digestion with modified trypsin (Promega). NanoLC-nanoESI-MS/MS analysis was performed on a nanoAcquity system (Waters) connected to the Orbitrap Elite hybrid mass spectrometer (Thermo Electron) equipped with a PicoView nanospray interface (New Objective) (Core Facilities for Protein Structural Analysis at the Institute of Biological Chemistry, Academia Sinica, Taiwan). The tryptic peptide mixtures were loaded onto a 75-μm x 250-mm nanoACQUITY UPLC BEH130 column packed with C18 resin (Waters, Milford USA) and separated at a flow rate of 300 nl/min using a gradient from 5% to 80% acetonitrile in 0.1% formic acid. The effluent from the HPLC column was directly electrosprayed into the mass spectrometer. The LTQ Orbitrap Velos instrument was operated in data-dependent mode to automatically switch between full scan MS and MS/MS acquisition. All data generated were searched against the NCBI database by the Mascot daemon software. Search criteria used were set as following: NCBI database; trypsin digestion; up to two missed cleavages allowed; variable modification set as carbamidomethyl (Cys), oxidation (Met), phosphorylation (Ser, Thr) and phosphorylation (Tyr); the mass accuracy is set as 10 ppm for precursor ion; peptide charge state set as 2+, 3+ and 4+. Phosphorylation sites and peptide sequence assignments contained in Mascot search results were validated by manual confirmation from raw MS/MS data.

## Supporting Information

S1 Fig The dendrite development is normal in *ik2* and *spn-F* mutant neurons. (A,B) The expression of *UAS-mCD8RFP* was driven by *ppk-GAL4* in C4da neurons of wild-type larvae (A), and in *ik2* mutant MARCM clone of ddaC neurons. (C, D) The expression of *ppk-eGFP* in the ddaC neurons of wild-type larvae (C), and of homozygous *spn-F* ^*2*^ mutant larvae. Scale bars, 50 μm.
(TIF)

S2 Fig The Spn-F-GFP distribution in larval and pupal ddaC neurons. (A and A’) The expression of *UAS-Spn-F-GFP* and *UAS-mCD8RFP* were driven by *ppk-GAL4* in C4da neurons. The punctate distribution of Spn-F-GFP was observed in the soma, dendrites (arrows), and axons (arrowheads) of ddaC neurons in larvae. (B-D) Live imaging of Spn-F-GFP distribution in the same ddaC neuron at larval stage (B) and at various time points of pupal stages (C, D). The fluorescent intensity of Spn-F-GFP signals along the line crossing the center of nucleus was plotted at various time points (E). The fluorescent intensity peaked as the line passing Spn-F-GFP puncta, and the rest was considered as dispersed GFP signals in the cytosol as indicated as an asterisk in (E). (F-I) The expression of *UAS-Spn-F-GFP* was controlled by *ppk-GAL4* in C4da neurons. (F'-I') The ddaC neurons were visualized with *ppk-GAL4* and *UAS-mCD8RFP*. The Spn-F-GFP showed punctate patterns in the cytosol of ddaC neurons of wild-type larvae (F), and became dispersed in larval neurons with wild-type *ik2* (*ik2-wt*) expression (G, H), but remained punctate in larval neurons with *ik2-G250D* expression (I). a.f.u.: arbitrary fluorescence units. Scale bars, 10 μm.
(TIF)

S3 Fig Spn-F was phosphorylated by Ik2 kinase in C4da neurons. Whole-cell lysates of control *w* ^*1118*^ larvae, of larvae with Spn-F-GFP expression in C4da neurons, and of larvae with Spn-F-GFP and wild-type Ik2 (or Ik2-G250D) expression in C4da neurons, were separated by SDS-PAGE using a gel containing Phos-tag acrylamide and blotted by antibodies against GFP. Phosphorylated Spn-F (P-Spn-F, indicated by an arrow) was detected only from the larval ddaC neurons with wild-type Ik2 expression, but not with Ik2-G250D expression or without Ik2 expression.
(JPG)

S4 Fig Label-free quantification of Spn-F phosphorylation percentage at S154, S172, S202 and S325 of Spn-F protein. Quantification of the TFTQHS^154^PNPHLR, GIKDLS^172^LEEIA, VEETTS^202^EPDAN and YSSQVS^325^FNAFR peptide ion signals based on calculated extracted ion chromatogram (XIC) area. Phosphorylation percentages of the S154, S172, S202 and S325 from S2 cells overexpressing Spn-F with Ik2 or Ik2-K41A are shown. S172, S202 and S325 of Spn-F show a higher phosphorylation level in S2 cells with Ik2 expression than that with Ik2-K41A expression.
(TIF)

S5 Fig Normal interaction between phospho-mutant Spn-F and Ik2 kinase. Co-IP was performed with lysates of S2 cells co-transfected with wild-type *spn-F*, *spnF-8A* or *spnF-8D* together with *ik2-FLAG* constructs, showing that SpnF-8A and -8D maintain normal interaction with Ik2, as the wild-type Spn-F does.
(TIF)

S6 Fig The formation of Spn-F puncta does not depend on intact microtubule cytoskeletons and the function of dynein complex. (A-C) The anti-tubulin Ab staining in S2 cells shows intact microtubule cytoskeletons in control cells (A) and in cells treated with (20 μM) ciliobrevin D (C), but becomes depolymerized in cells treated with (10 μM) colchicine (B). The Spn-F puncta were formed normally in S2 cells treated with colchicine (D) and with ciliobrevin D (E). Scale bar, 10 μm.
(TIF)

S7 Fig The dendrite development is normal in *Dhc* mutant neurons. (A-C) The expression of *UAS-mCD8RFP* was driven by *ppk-GAL4* in C4da neurons of wild-type (A), *Dhc* ^*6-6*^/*Dhc* ^*6-10*^ (B), and of *Dhc* ^*6-6*^/*Df* (C) larvae. Scale bars, 50 μm.
(TIF)

S8 Fig The immunofluorescent staining of Ik2, Ctp and Spn-F proteins in S2 cells. (A) Both Ik2-HA and Ctp-FLAG signals were dispersed in the cytosol of transfected S2 cells. In addition to the cytosol, Ctp-FLAG signals were also detected in the nucleus of transfected S2 cells. (B) The dispersion of Ik2-HA, Spn-F-GFP and Ctp-FLAG were observed in the cytosol of S2 cells co-expressing all three molecules. Scale bar, 10 μm.
(TIF)

S9 Fig SpnF-8A and SpnF-8D retain normal interaction with Ik2 and Ctp. (A) Co-IP assays were performed with lysates from S2 cells transfected with SpnF-myc, SpnF-8A-myc or SpnF-8D-myc, Ctp-FLAG and Ik2-HA or Ik2-G250D-HA to show that the formation of Ik2 (or Ik2-G250D)/Spn-F (or SpnF-8A or SpnF-8D)/Ctp tertiary complexes depends on the presence of Spn-F proteins. (B) Quantitative analysis of colocalization of Ik2-G250D/Ctp, Ik2-G250D/Spn-F/Ctp, Ik2-G250D/SpnF-8A/Ctp, and Ik2-G250D/SpnF-8D/Ctp in transfected S2 cells. The percentage of S2 cells shows colocalization of staining signals among the total number of examined cells that expressed all transfected proteins. The percentages of colocalization were calculated from three independent experiments: for Ik2-G250D/Ctp transfected cells, n = 10/95, 6/103 and 6/103; for Ik2-G250D/Spn-F/Ctp transfected cells, n = 100/110, 100/102 and 106/109; for Ik2-G250D/SpnF-8A/Ctp transfected cells, n = 88/106, 100/108 and 100/107; for Ik2-G250D/SpnF-8D/Ctp transfected cells, n = 94/103, 97/105 and 99/100. Error bars show SD.
(TIF)

S10 Fig The sequence alignment of Spn-F SCD domain and the deletion constructs of Spn-F. (A) The amino acid sequence alignment between the Spn-F C-terminal domain (SCD) from *Drosophila melanogaster* and that from different insect species, including *Andes aegypti* (mosquito), *Bombyx mori* (silkworm), *Tribolium castaneum* (beetle), *Apis mellifera* (honey bee), and *Nasonia vitripennis* (wasp). The sequence homology of Spn-F homologues among species was shown in gray boxes. The residue numbers of Spn-F from *D*. *melanogaster* were indicated on the top. The sequences with underline were deleted to generate the SpnF-ΔSCD mutant in (B). (B) A schematic shows various Spn-F deletion protein constructs. The Spn-F proteins have three coiled-coil domains (CC1, CC2 and CC3), and a SpnF-conserved domain (SCD), which is highly conserved among different insect species, at its carboxyl terminus.
(TIF)

S11 Fig SpnF-ΔCC3 retains normal self-interaction with SpnF-ΔCC3 and full-length Spn-F. Co-IP was performed with lysates of S2 cells cotransfected with *spnF*-ΔCC3 only or spnF-ΔCC3 and full-length Spn-F, showing that SpnF-ΔCC3 retains normal self-interaction with SpnF-ΔCC3 and full-length Spn-F.
(TIF)

S12 Fig The immunostaining of P-Ik2 in *Dhc* mutant neurons, and dendrite pruning defects in *Dhc spn-F* double mutants and *ik2 Dhc* double mutants. (A-C) The activated Ik2 signals were detected by antibodies against phosphorylated Ik2 (P-Ik2) in larvae and pupae at 1 h APF. (A'-C') The ddaC neurons were visualized with *ppk-GAL4* and *UAS-mCD8RFP*. The strong P-Ik2 signals were observed in wild-type (B) and *Dhc*-RNAi (C) pupal C4da neurons at 1 h APF, but not in wild-type larval neurons (A). (D,E) Quantitative analysis of dendrite pruning phenotypes in ddaC cells at 16 h APF. The percentage of ddaC neurons shows dendrite pruning defects among the total number of neurons examined. (D) For wild-type control, n = 50; for *Dhc-RNAi*, n = 70; for *spn-F* ^*2*^ mutants, n = 90; for *Dhc-RNAi* in *spn-F* ^*2*^ mutants, n = 30. (E) For wild-type control, n = 50; for *ik2-RNAi*, n = 100; for *Dhc* ^*6-6*^/*Df* mutants, n = 90; for *ik2-RNAi* in *Dhc* ^*6-6*^/*Df* mutants, n = 70. Statistical analysis was performed with Chi-square test. N.S., not significant. Scale bar, 10 μm.
(TIF)

S13 Fig The local breakage of microtubules in the proximal dendrites of C4da neurons was suppressed in *spn-F* RNAi mutants. The immunostaining with anti-GFP Abs was used to detect microtubules (*UAS-tub-GFP* expression under *ppk-GAL4*) in wild-type (A) and in *spn-F* RNAi (B) ddaC neurons at 5 h APF, suggesting that *spn-F*, like *ik2*, plays a role in dendrite severing that involves local microtubule disassembly. Scale bar, 50 μm.
(TIF)

S1 Table Spn-F phosphorylation sites identified by LC-MS/MS from S2 cells. (PDF)

S2 Table Identification of Spn-F phosphorylation sites. (PDF)

S1 Movie The redistribution of Spn-F in pupal ddaC neurons. Spn-F-GFP expression was directed by *ppk-GAL4* in C4da neurons, and the movie was taken to record the distribution of Spn-F-GFP in the same ddaC cells from 0 to 2 h APF.
(MP4)

S2 Movie The redistribution of Spn-F in pupal ddaC neurons. Spn-F-GFP expression was directed by *ppk-GAL4* in C4da neurons, and the movie was taken to record the distribution of Spn-F-GFP in the same ddaC cells from 0.5 to 1.58 h APF.
(MP4)
