# Supplementary figures and images for: Spindle-F Is the Central Mediator of Ik2 Kinase-Dependent Dendrite Pruning in Drosophila Sensory Neurons
Source: PLoS Genet. 2015 Nov 5;11(11):e1005642. doi: 10.1371/journal.pgen.1005642 (PMC4634852; doi:10.1371/journal.pgen.1005642)

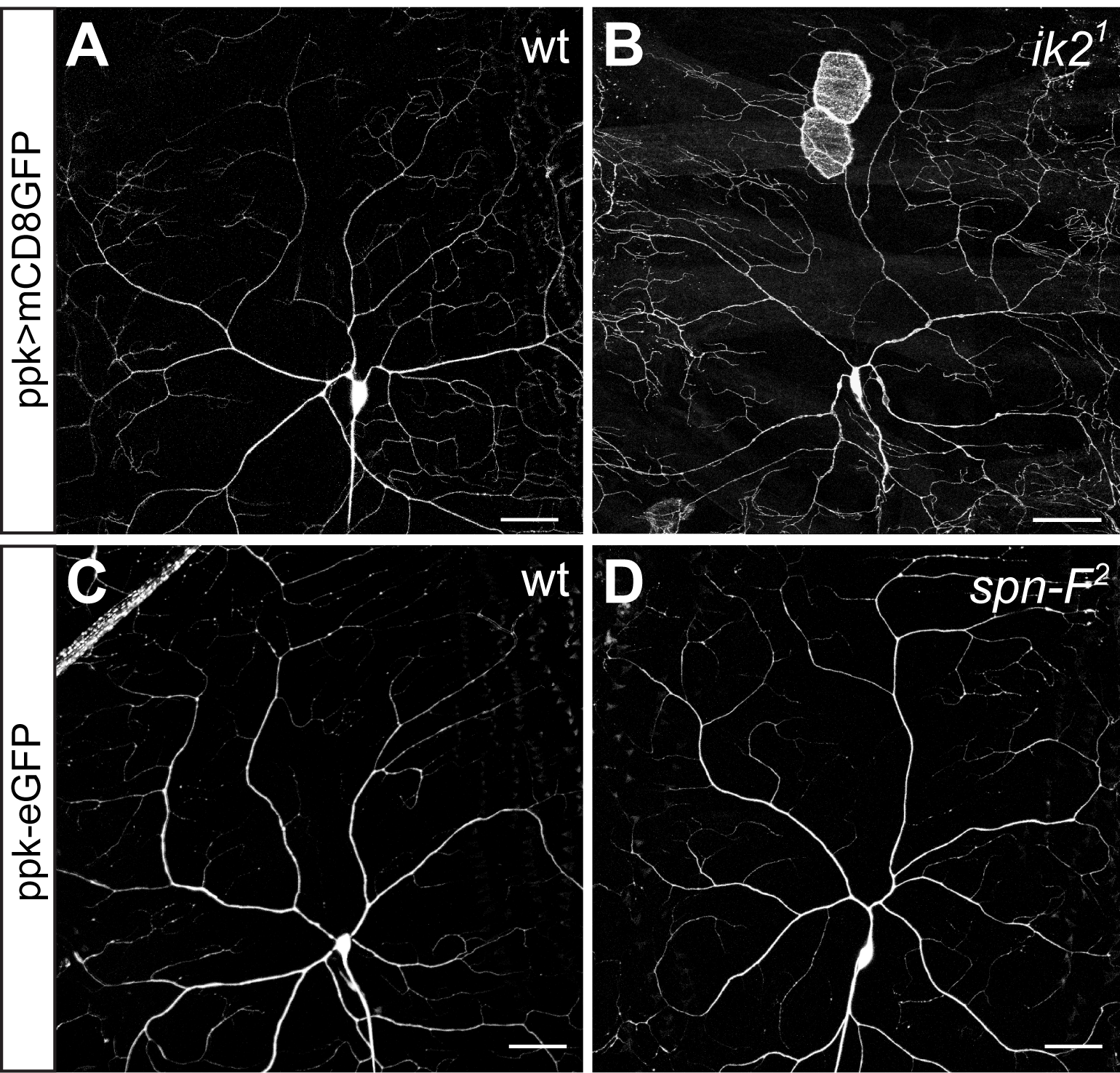

Supplement: S1 Fig — (A,B) The expression of UAS-mCD8RFP was driven by ppk-GAL4 in C4da neurons of wild-type larvae (A), and in ik2 mutant MARCM clone of ddaC neurons. (C, D) The expression of ppk-eGFP in the ddaC neurons of wild-type larvae (C), and of homozygous spn-F 2 mutant larvae. Scale bars, 50 μm. (TIF) [file pgen.1005642.s001.tif]

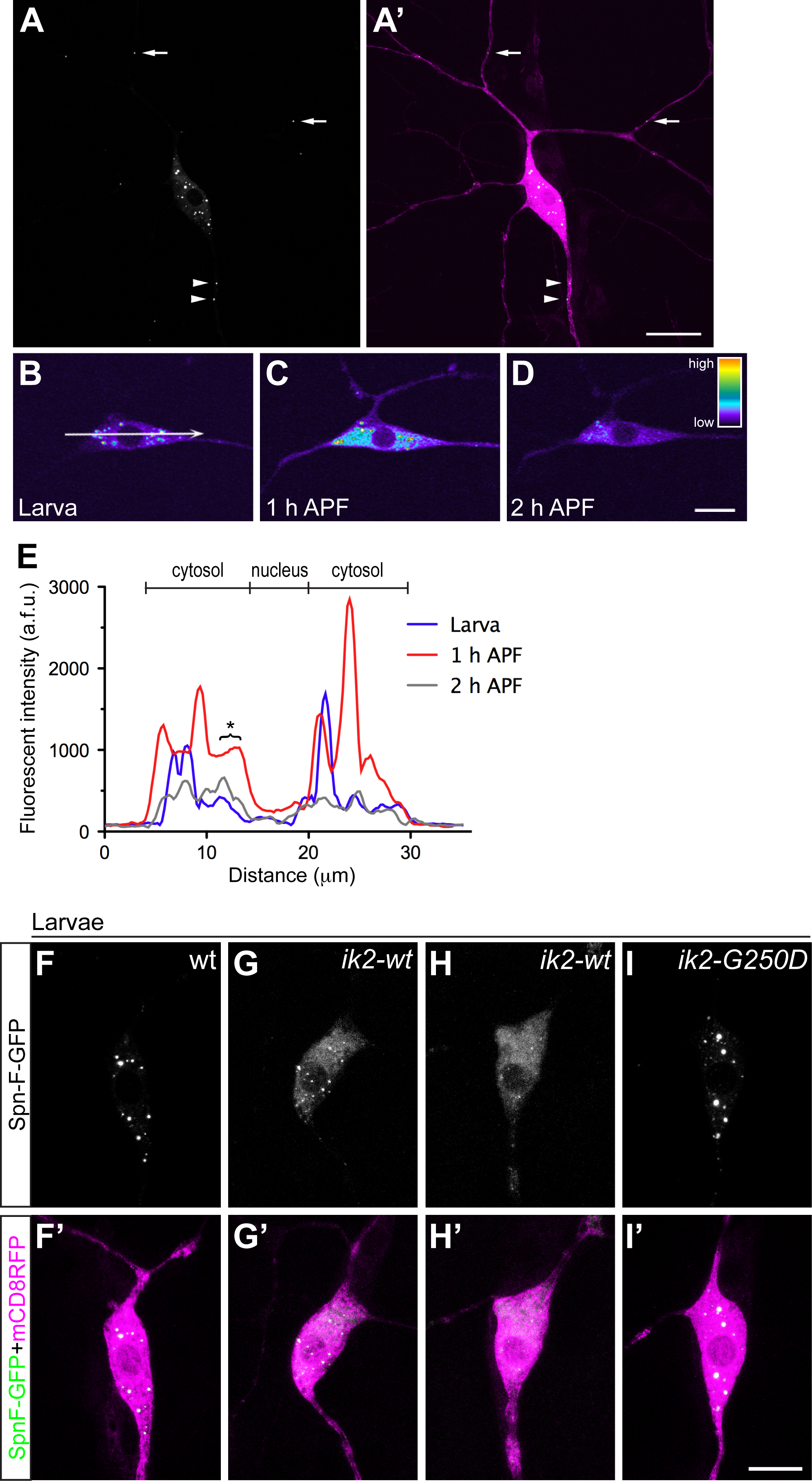

Supplement: S2 Fig — (A and A’) The expression of UAS-Spn-F-GFP and UAS-mCD8RFP were driven by ppk-GAL4 in C4da neurons. The punctate distribution of Spn-F-GFP was observed in the soma, dendrites (arrows), and axons (arrowheads) of ddaC neurons in larvae. (B-D) Live imaging of Spn-F-GFP distribution in the same ddaC neuron at larval stage (B) and at various time points of pupal stages (C, D). The fluorescent intensity of Spn-F-GFP signals along the line crossing the center of nucleus was plotted at various time points (E). The fluorescent intensity peaked as the line passing Spn-F-GFP puncta, and the rest was considered as dispersed GFP signals in the cytosol as indicated as an asterisk in (E). (F-I) The expression of UAS-Spn-F-GFP was controlled by ppk-GAL4 in C4da neurons. (F'-I') The ddaC neurons were visualized with ppk-GAL4 and UAS-mCD8RFP. The Spn-F-GFP showed punctate patterns in the cytosol of ddaC neurons of wild-type larvae (F), and became dispersed in larval neurons with wild-type ik2 (ik2-wt) expression (G, H), but remained punctate in larval neurons with ik2-G250D expression (I). a.f.u.: arbitrary fluorescence units. Scale bars, 10 μm. (TIF) [file pgen.1005642.s002.tif]

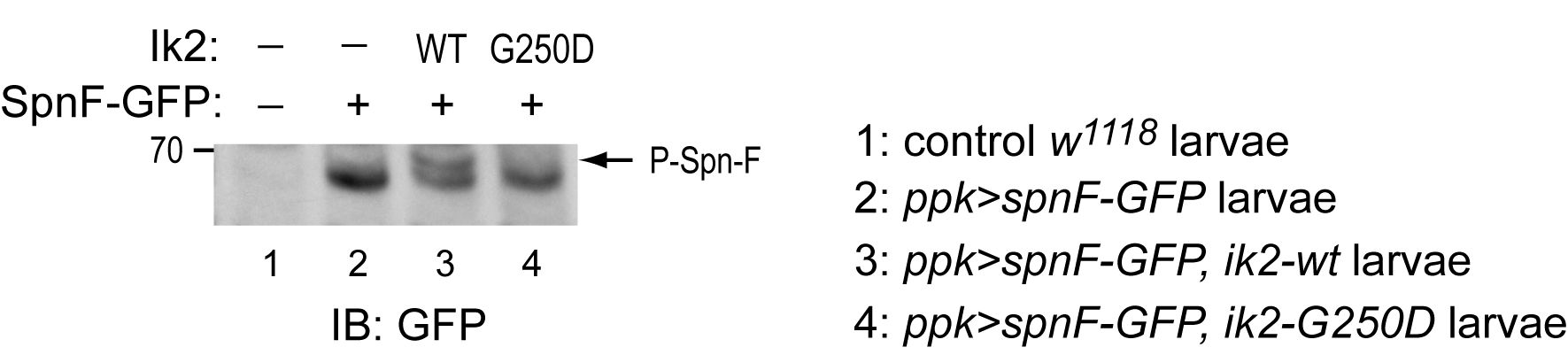

Supplement: S3 Fig — Whole-cell lysates of control w 1118 larvae, of larvae with Spn-F-GFP expression in C4da neurons, and of larvae with Spn-F-GFP and wild-type Ik2 (or Ik2-G250D) expression in C4da neurons, were separated by SDS-PAGE using a gel containing Phos-tag acrylamide and blotted by antibodies against GFP. Phosphorylated Spn-F (P-Spn-F, indicated by an arrow) was detected only from the larval ddaC neurons with wild-type Ik2 expression, but not with Ik2-G250D expression or without Ik2 expression. (JPG) [file pgen.1005642.s003.jpg]

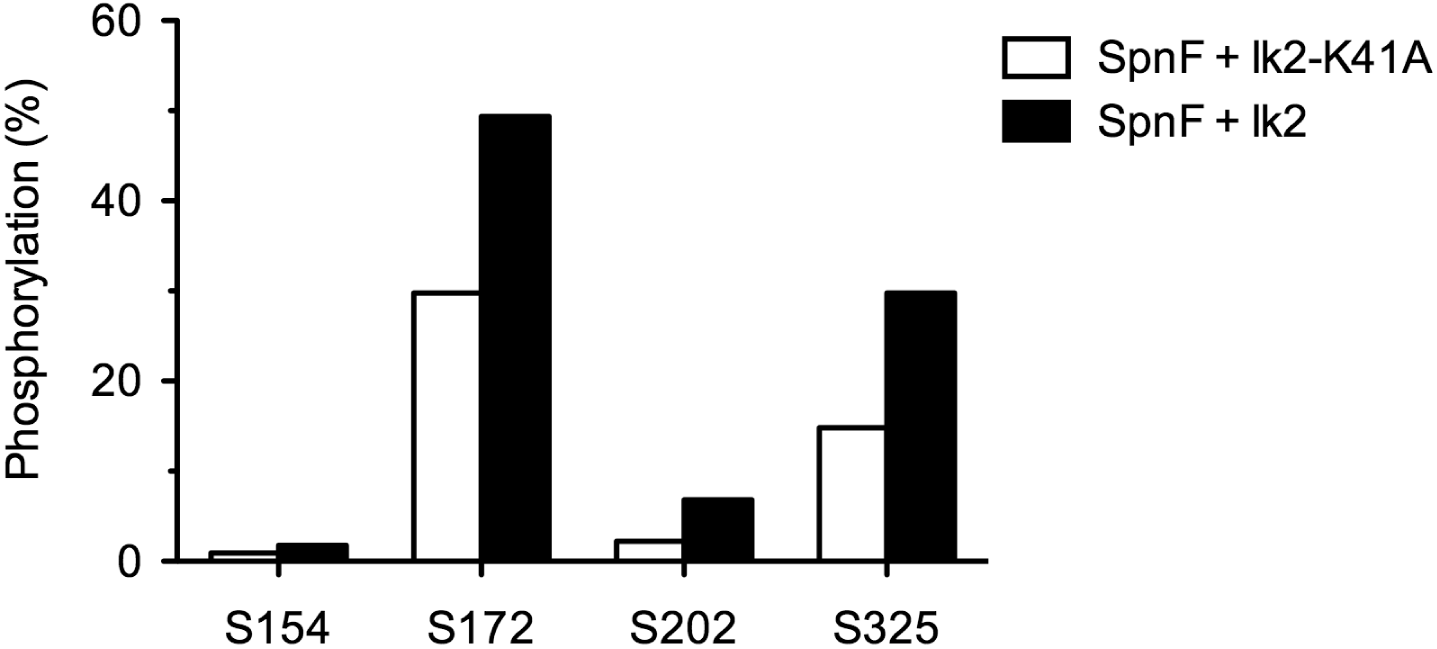

Supplement: S4 Fig — Quantification of the TFTQHS154PNPHLR, GIKDLS172LEEIA, VEETTS202EPDAN and YSSQVS325FNAFR peptide ion signals based on calculated extracted ion chromatogram (XIC) area. Phosphorylation percentages of the S154, S172, S202 and S325 from S2 cells overexpressing Spn-F with Ik2 or Ik2-K41A are shown. S172, S202 and S325 of Spn-F show a higher phosphorylation level in S2 cells with Ik2 expression than that with Ik2-K41A expression. (TIF) [file pgen.1005642.s004.tif]

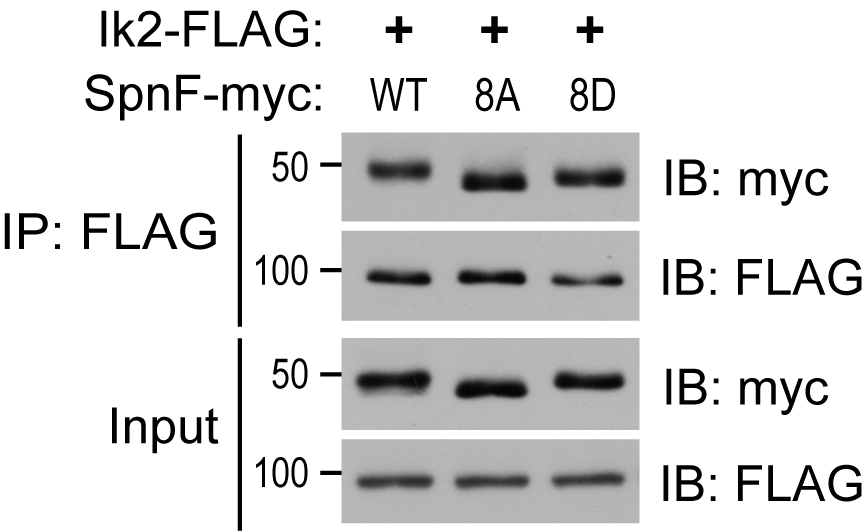

Supplement: S5 Fig — Co-IP was performed with lysates of S2 cells co-transfected with wild-type spn-F, spnF-8A or spnF-8D together with ik2-FLAG constructs, showing that SpnF-8A and -8D maintain normal interaction with Ik2, as the wild-type Spn-F does. (TIF) [file pgen.1005642.s005.tif]

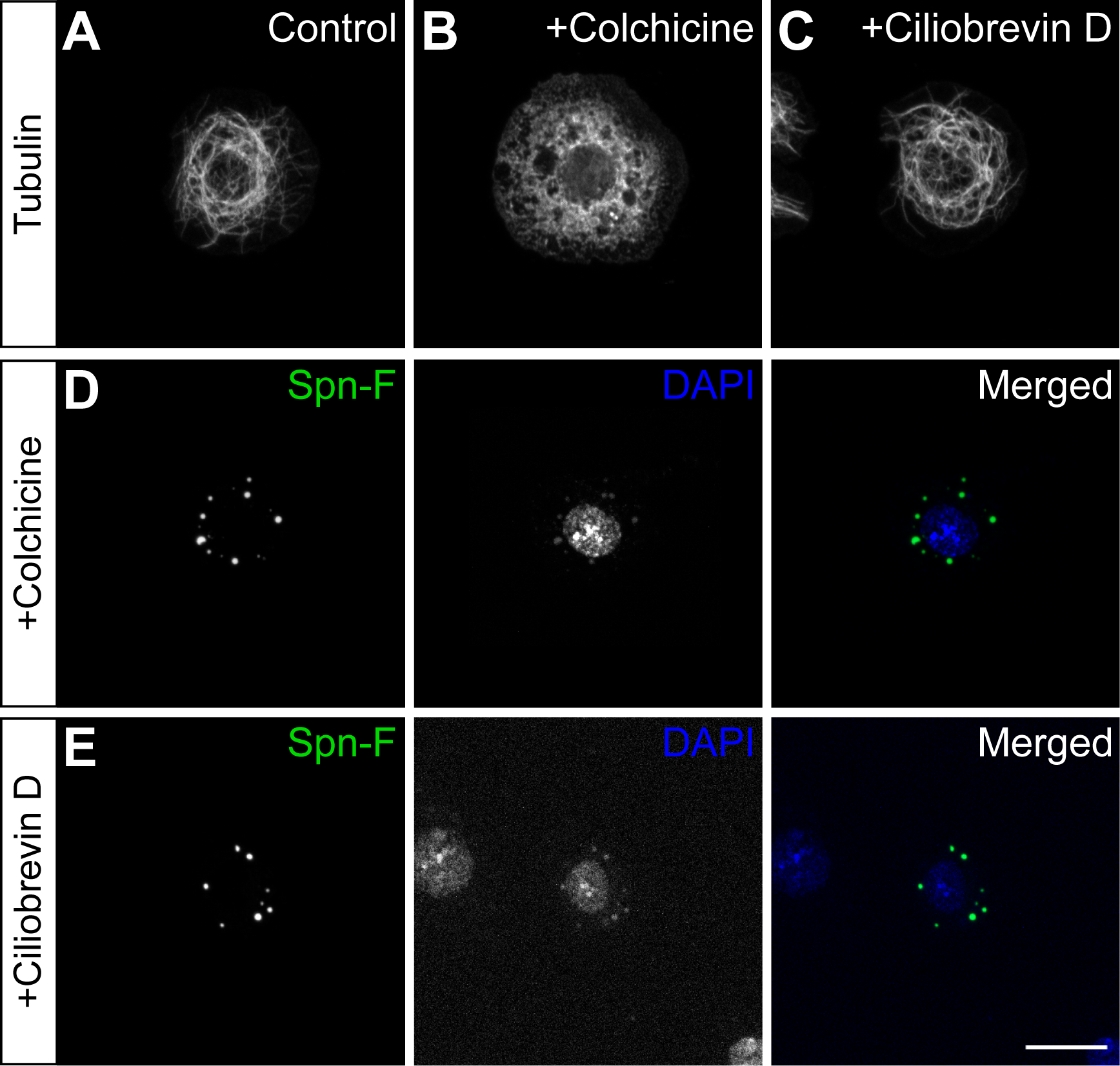

Supplement: S6 Fig — (A-C) The anti-tubulin Ab staining in S2 cells shows intact microtubule cytoskeletons in control cells (A) and in cells treated with (20 μM) ciliobrevin D (C), but becomes depolymerized in cells treated with (10 μM) colchicine (B). The Spn-F puncta were formed normally in S2 cells treated with colchicine (D) and with ciliobrevin D (E). Scale bar, 10 μm. (TIF) [file pgen.1005642.s006.tif]

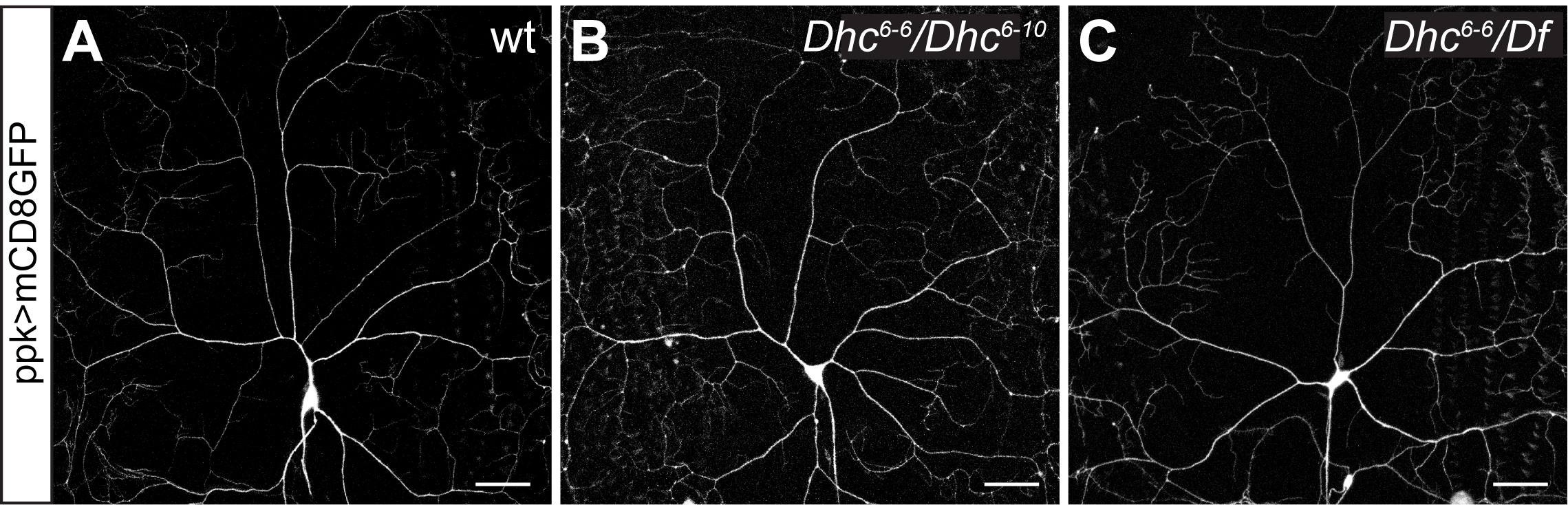

Supplement: S7 Fig — (A-C) The expression of UAS-mCD8RFP was driven by ppk-GAL4 in C4da neurons of wild-type (A), Dhc 6-6/Dhc 6-10 (B), and of Dhc 6-6/Df (C) larvae. Scale bars, 50 μm. (TIF) [file pgen.1005642.s007.tif]

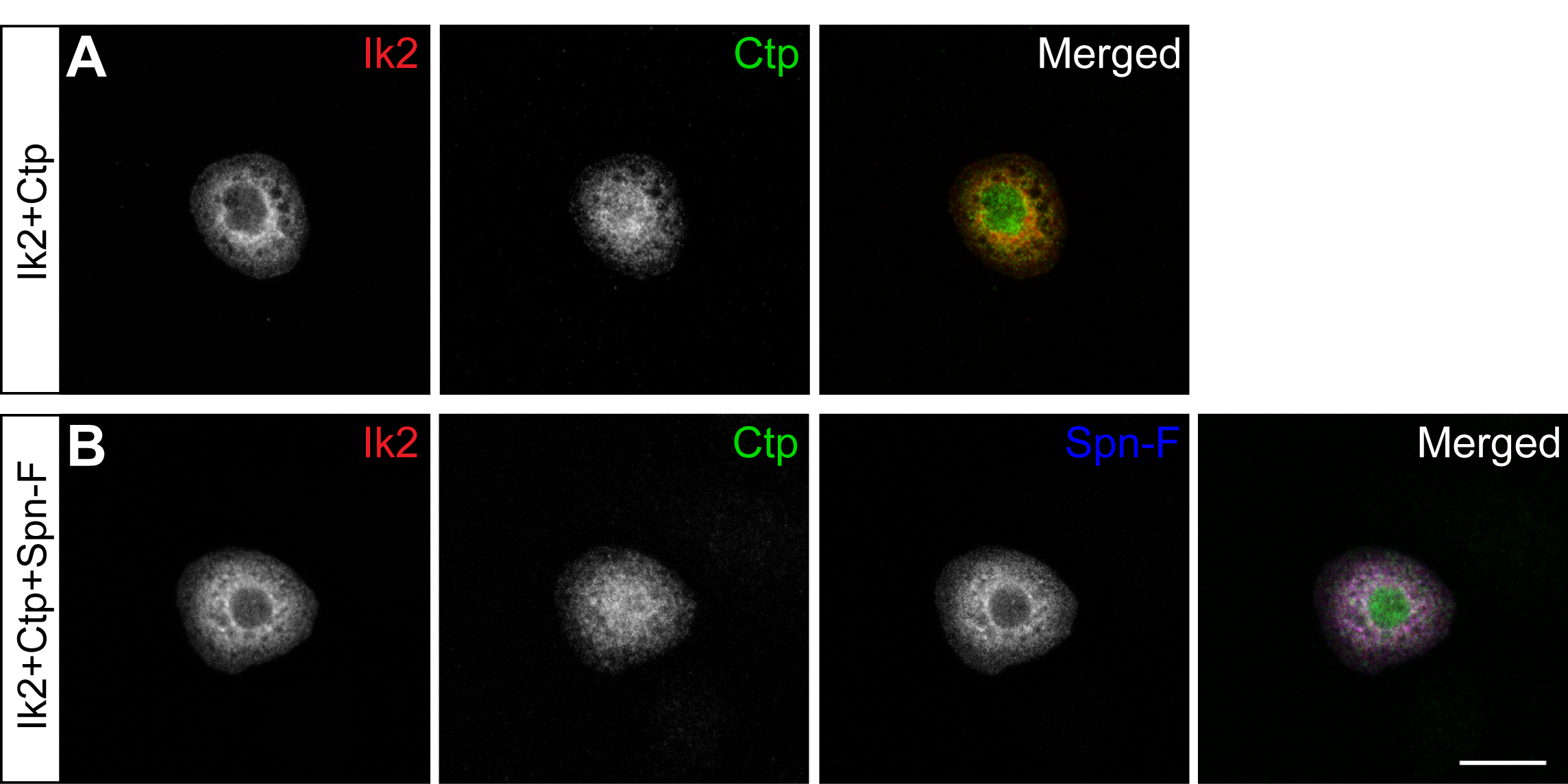

Supplement: S8 Fig — (A) Both Ik2-HA and Ctp-FLAG signals were dispersed in the cytosol of transfected S2 cells. In addition to the cytosol, Ctp-FLAG signals were also detected in the nucleus of transfected S2 cells. (B) The dispersion of Ik2-HA, Spn-F-GFP and Ctp-FLAG were observed in the cytosol of S2 cells co-expressing all three molecules. Scale bar, 10 μm. (TIF) [file pgen.1005642.s008.tif]

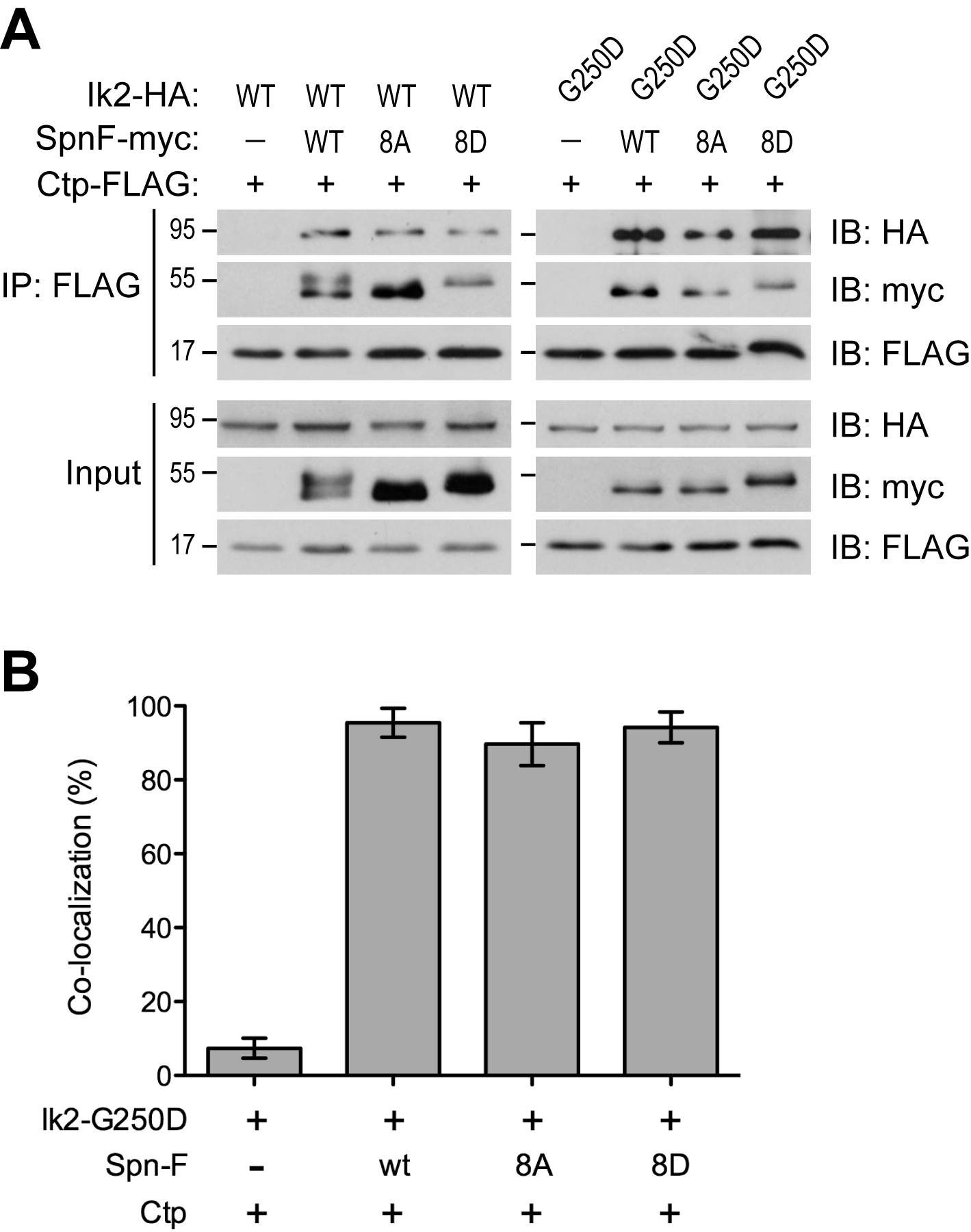

Supplement: S9 Fig — (A) Co-IP assays were performed with lysates from S2 cells transfected with SpnF-myc, SpnF-8A-myc or SpnF-8D-myc, Ctp-FLAG and Ik2-HA or Ik2-G250D-HA to show that the formation of Ik2 (or Ik2-G250D)/Spn-F (or SpnF-8A or SpnF-8D)/Ctp tertiary complexes depends on the presence of Spn-F proteins. (B) Quantitative analysis of colocalization of Ik2-G250D/Ctp, Ik2-G250D/Spn-F/Ctp, Ik2-G250D/SpnF-8A/Ctp, and Ik2-G250D/SpnF-8D/Ctp in transfected S2 cells. The percentage of S2 cells shows colocalization of staining signals among the total number of examined cells that expressed all transfected proteins. The percentages of colocalization were calculated from three independent experiments: for Ik2-G250D/Ctp transfected cells, n = 10/95, 6/103 and 6/103; for Ik2-G250D/Spn-F/Ctp transfected cells, n = 100/110, 100/102 and 106/109; for Ik2-G250D/SpnF-8A/Ctp transfected cells, n = 88/106, 100/108 and 100/107; for Ik2-G250D/SpnF-8D/Ctp transfected cells, n = 94/103, 97/105 and 99/100. Error bars show SD. (TIF) [file pgen.1005642.s009.tif]

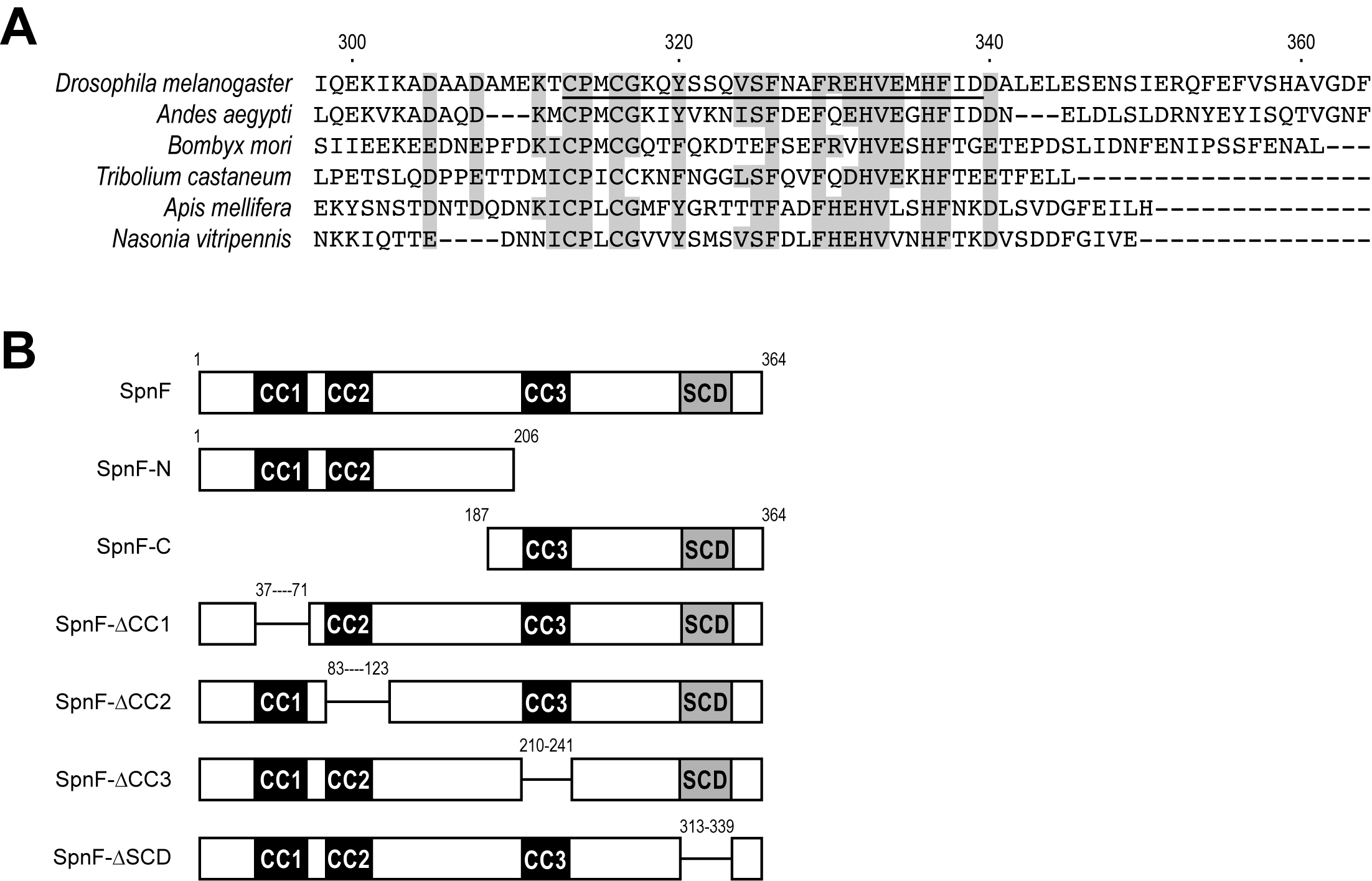

Supplement: S10 Fig — (A) The amino acid sequence alignment between the Spn-F C-terminal domain (SCD) from Drosophila melanogaster and that from different insect species, including Andes aegypti (mosquito), Bombyx mori (silkworm), Tribolium castaneum (beetle), Apis mellifera (honey bee), and Nasonia vitripennis (wasp). The sequence homology of Spn-F homologues among species was shown in gray boxes. The residue numbers of Spn-F from D. melanogaster were indicated on the top. The sequences with underline were deleted to generate the SpnF-ΔSCD mutant in (B). (B) A schematic shows various Spn-F deletion protein constructs. The Spn-F proteins have three coiled-coil domains (CC1, CC2 and CC3), and a SpnF-conserved domain (SCD), which is highly conserved among different insect species, at its carboxyl terminus. (TIF) [file pgen.1005642.s010.tif]

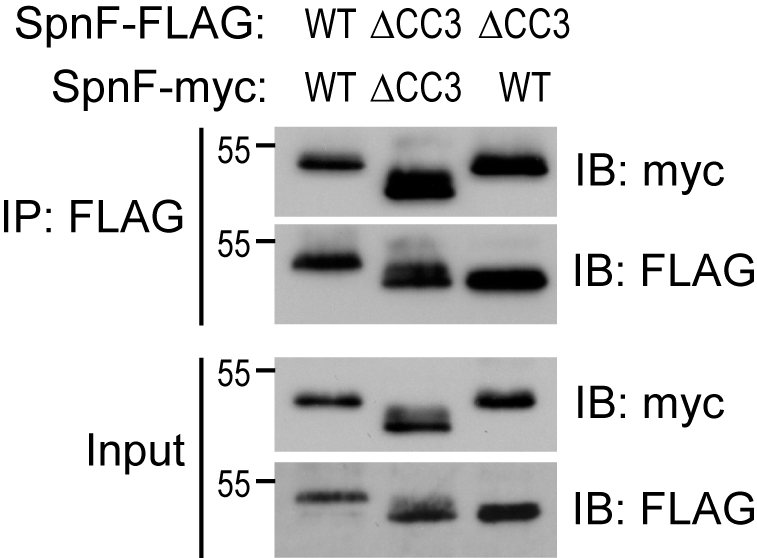

Supplement: S11 Fig — Co-IP was performed with lysates of S2 cells cotransfected with spnF-ΔCC3 only or spnF-ΔCC3 and full-length Spn-F, showing that SpnF-ΔCC3 retains normal self-interaction with SpnF-ΔCC3 and full-length Spn-F. (TIF) [file pgen.1005642.s011.tif]

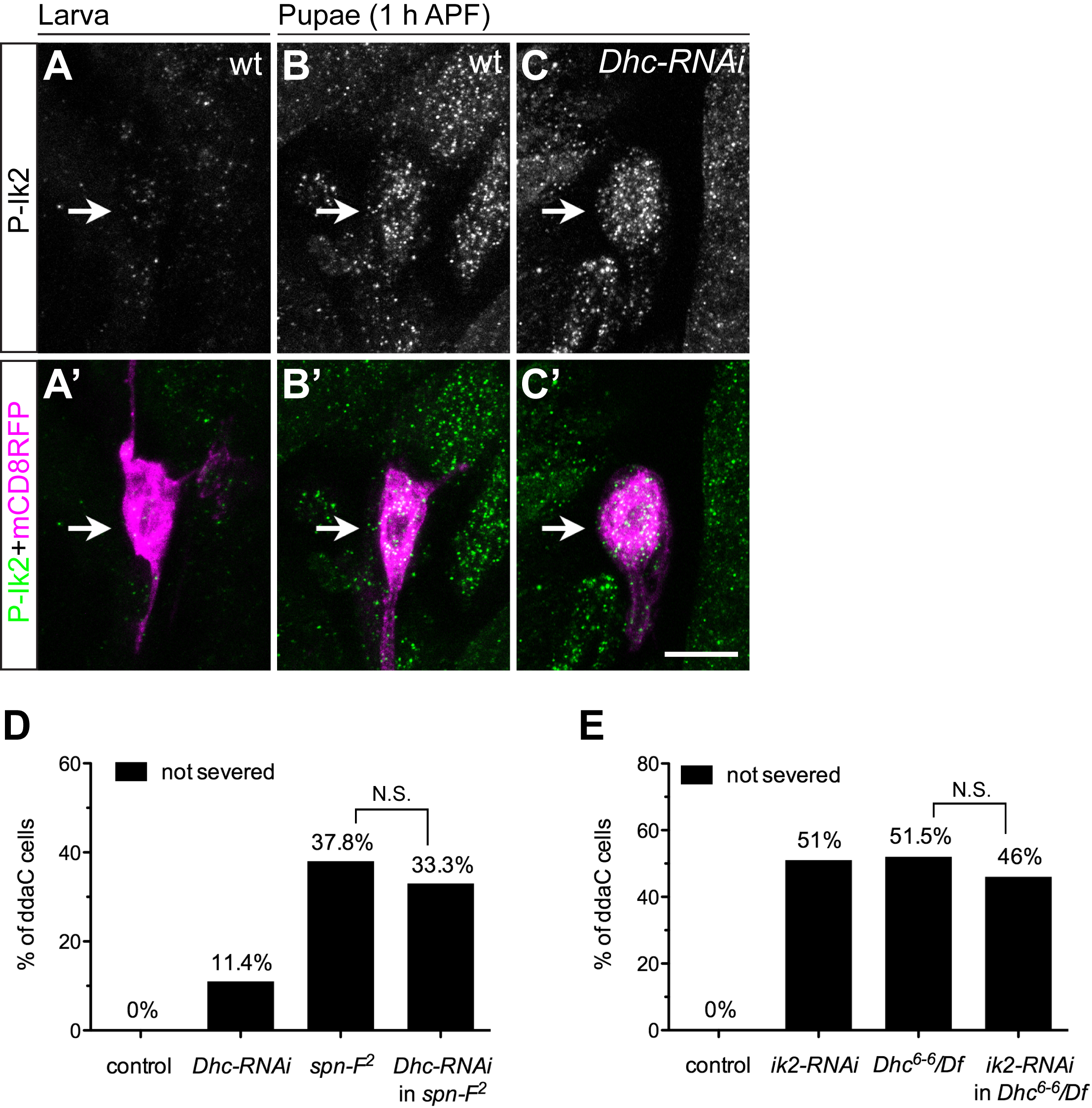

Supplement: S12 Fig — (A-C) The activated Ik2 signals were detected by antibodies against phosphorylated Ik2 (P-Ik2) in larvae and pupae at 1 h APF. (A'-C') The ddaC neurons were visualized with ppk-GAL4 and UAS-mCD8RFP. The strong P-Ik2 signals were observed in wild-type (B) and Dhc-RNAi (C) pupal C4da neurons at 1 h APF, but not in wild-type larval neurons (A). (D,E) Quantitative analysis of dendrite pruning phenotypes in ddaC cells at 16 h APF. The percentage of ddaC neurons shows dendrite pruning defects among the total number of neurons examined. (D) For wild-type control, n = 50; for Dhc-RNAi, n = 70; for spn-F 2 mutants, n = 90; for Dhc-RNAi in spn-F 2 mutants, n = 30. (E) For wild-type control, n = 50; for ik2-RNAi, n = 100; for Dhc 6-6/Df mutants, n = 90; for ik2-RNAi in Dhc 6-6/Df mutants, n = 70. Statistical analysis was performed with Chi-square test. N.S., not significant. Scale bar, 10 μm. (TIF) [file pgen.1005642.s012.tif]

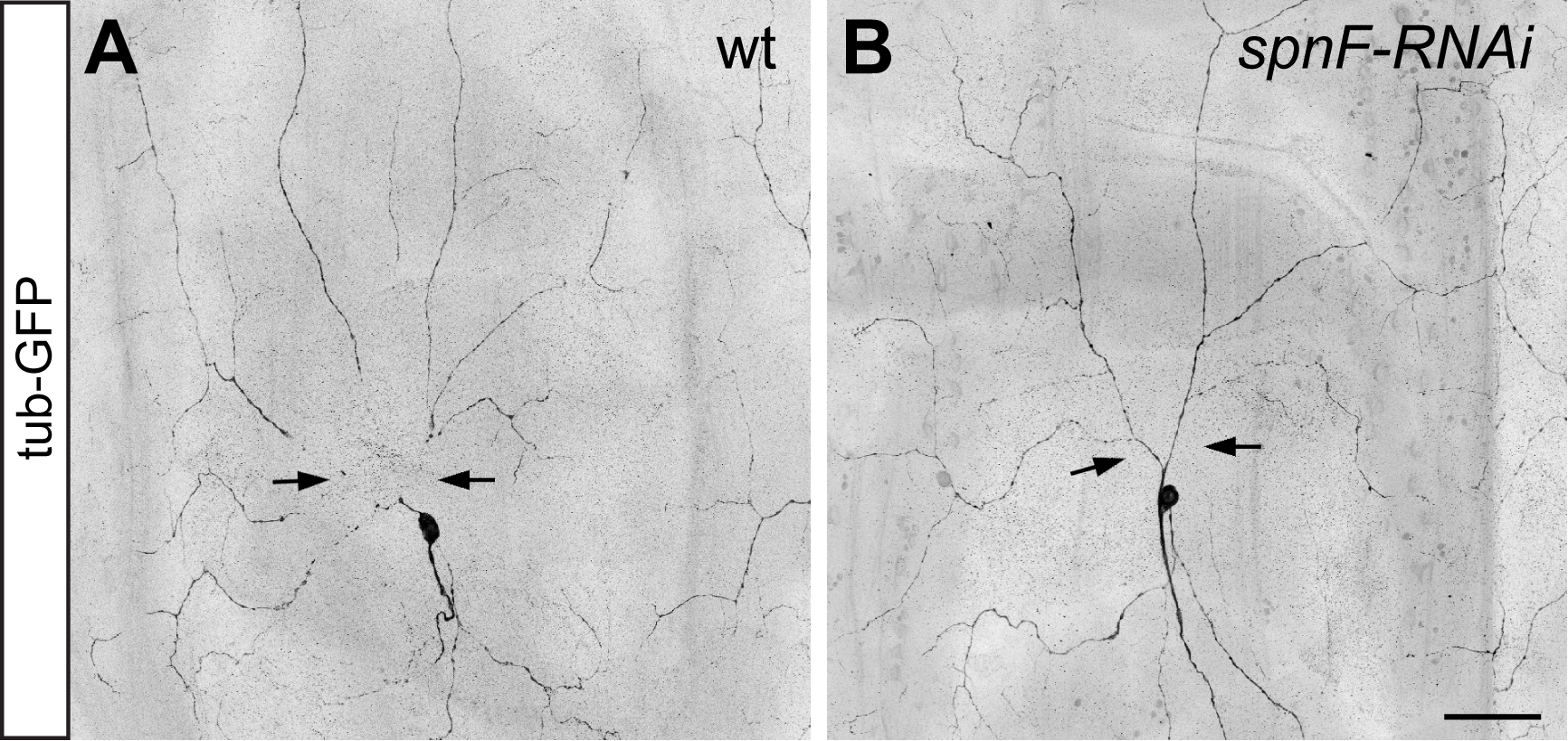

Supplement: S13 Fig — The immunostaining with anti-GFP Abs was used to detect microtubules (UAS-tub-GFP expression under ppk-GAL4) in wild-type (A) and in spn-F RNAi (B) ddaC neurons at 5 h APF, suggesting that spn-F, like ik2, plays a role in dendrite severing that involves local microtubule disassembly. Scale bar, 50 μm. (TIF) [file pgen.1005642.s013.tif]
