# Supplementary material for: Spindle-F Is the Central Mediator of Ik2 Kinase-Dependent Dendrite Pruning in Drosophila Sensory Neurons
Source: PLoS Genet. 2015 Nov 5;11(11):e1005642. doi: 10.1371/journal.pgen.1005642 (PMC4634852; doi:10.1371/journal.pgen.1005642)
Supplement: S1 Table — (PDF) [file pgen.1005642.s014.pdf]

**Table 1. Spn-F phosphorylation sites identified by LC-MS/MS from S2 cells.**

| Phosphorylated Residue | Phosphopeptide sequence | Mascot Ion Score | Mascot expectation value | Charge | m/z |
|---|---|---|---|---|---|
| S53 | VA**Sp**MEEENQQLR | 42.3 | $1.1x10^{-2}$ | 2 | 765.3183 |
| S85 | ANEIGVTGDVL**Sp**LK | 60.8 | $1.7x10^{-4}$ | 2 | 748.3746 |
| S172 | DL**Sp**LEEIALDDFGASSEELGYPYNLQK | 25.6 | 1.5 | 3 | 1032.8010 |
| S202 | VEETT**Sp**EPDANVDAK | 58.5 | $3.1x10^{-4}$ | 2 | 842.8511 |
| S264 | KPEMADK**Sp**LETDDSLTSELK | 42.5 | $2.1\ x10^{-2}$ | 3 | 773.0220 |
| S270 | SLETDD**Sp**LTSELK | 44.4 | $6.9\ x10^{-3}$ | 2 | 759.3357 |
| S325 | QYSSQV**Sp**FNAFR | 42.0 | $1.2\ x10^{-2}$ | 2 | 757.3266 |
| S349 | EHVEMHFIDDALELESEN**Sp**IER | 50.2 | $4.1\ x10^{-3}$ | 3 | 913.3936 |

Phosphorylated peptides were identified by combined one-dimensional capillary liquid chromatography (LC) – mass spectrometry and tandem mass spectrometry analysis.

Residues marked with "**p**" are Spn-F phosphorylation sites.

m/z, atomic mass units (amu).
