# Supplementary material for: Spindle-F Is the Central Mediator of Ik2 Kinase-Dependent Dendrite Pruning in Drosophila Sensory Neurons
Source: PLoS Genet. 2015 Nov 5;11(11):e1005642. doi: 10.1371/journal.pgen.1005642 (PMC4634852; doi:10.1371/journal.pgen.1005642)
Supplement: S2 Table — (PDF) [file pgen.1005642.s015.pdf]

Table 2. Identification of Spn-F phosphorylation sites

Residue modified	Mass result 1		Mass result 2	
	Spn-F and Ik2-K41A	Spn-F and Ik2	Spn-F	Spn-F and Ik2
Ser-23	X		X	X
Ser-53*		X		X
Ser-85*		X		X
Ser-91	X			
Ser-119			X	
Ser-154	X	X	X	X
Ser-165				X
Ser-172	X	X	X	X
Ser-202	X	X	X	X
Ser-264*		X		X
Ser-270*		X		X
Ser-325	X	X		X
Ser-349*		X		X

Residues marked with an "X" are phosphorylated and detected by Lc-MS/MS. Ser-53*, Ser-85*, Ser-264*, Ser-270* and Ser-349* of Spn-F are phosphorylated in S2 cells with Spn-F and Ik2 co-expression, but not with Spn-F only or not with Spn-F and Ik2-K41A co-expression. Additionally, Ser-154, Ser-172, Ser-202 and Ser-325 are phosphorylated in both experiments.
